# Reactions of 3-(*p*-substituted-phenyl)-5-chloromethyl-1,2,4-oxadiazoles with KCN leading to acetonitriles and alkanes via a non-reductive decyanation pathway

**DOI:** 10.3762/bjoc.14.280

**Published:** 2018-12-10

**Authors:** Akın Sağırlı, Yaşar Dürüst

**Affiliations:** 1 Department of Chemistry, Faculty of Arts & Sciences, Bolu Abant Izzet Baysal University, Bolu, TR14030, Turkey

**Keywords:** decyanation, KCN, 1,2,4-oxadiazole

## Abstract

The present work describes an unfamiliar reaction of 5-(chloromethyl)-3-substituted-phenyl-1,2,4-oxadiazoles with KCN affording trisubstituted 1,2,4-oxadiazol-5-ylacetonitriles and their parent alkanes, namely, 1,2,3-trisubstituted-1,2,4-oxadiazol-5-ylpropanes. To the best of our knowledge, the current synthetic route leading to decyanated products will be the first in terms of a decyanation process which allows the transformation of trisubstituted acetonitriles into alkanes by the incorporation of KCN with the association of in situ-formed HCN and most likely through the extrusion of cyanogen which could not be detected or isolated. In addition, the plausible mechanisms were proposed for both transformations. The structures of the title compounds were identified by means of IR, ^1^H NMR, ^13^C NMR, 2D NMR spectra, TOF–MS and X-ray measurements.

## Introduction

Heterocyclic scaffolds bearing 1,2,4-oxadiazole rings have been the subject of an increasing and remarkable attention due to their various bioactivities, such as anticancer [1], antimicrobial [2], antifungal [3], anti-inflammatory [4], tyrosine kinase inhibition [5] and histamine H3 antagonism properties [6]. In addition, these five-membered heterocycles were widely used as components of organic light emitting diodes (OLEDs), polymers, liquid crystals, and solar cells [7–10]. Taking into account the above considerations, new synthetic protocols to develop 1,2,4-oxadiazole-based heterocycles have gained an increasing attention over the recent decades [11–13].

**Figure 1:**
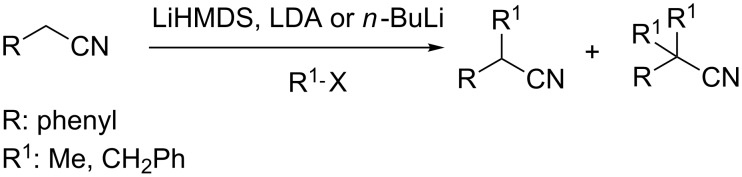
Synthesis of mono- or dialkylated acetonitriles.

On the other hand, arylacetonitriles are known as valuable intermediates that are generally obtained by the reaction of benzyl halides with appropriate cyanating agents such as KCN [14], TMSCN [15], K_4_(Fe(CN)_6_ [16]. Deprotonation of the α-carbon (adjacent to nitrile) by strong bases, especially lithiated ones, resulted in an anionic species that easily undergoes a substitution reaction with various alkyl halides to afford mono-, di- or trialkylated acetonitriles [17]. Most recently, Strzalko and co-workers disclose mono and dialkylation of the benzylic carbon of phenylacetonitrile with a poor selectivity by benzyl and methyl halides in the presence of LiHMDS, LDA or *n*-BuLi (Figure 1) [18]. However, such alkylation methods need harsh reaction conditions, most particularly lithiated bases and inert atmosphere.

**Figure 2:**
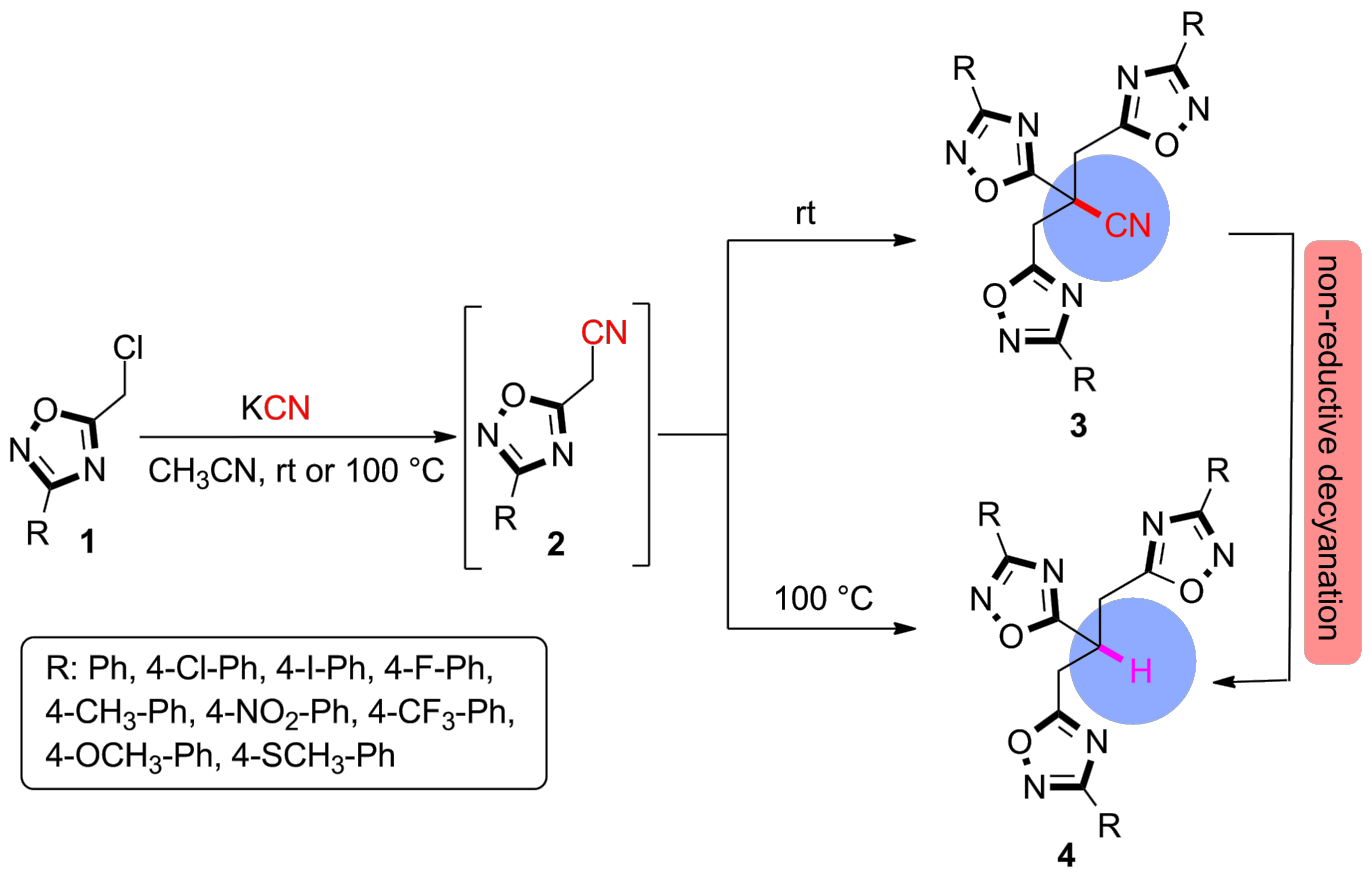
Cyanation through 5-chloromethyl-3-(*p*-substituted-phenyl)-1,2,4-oxadiazole.

In order to get a deeper understanding of the limitation step of alkylation processes and to extend their scope in the synthesis of mono-, di- or trialkylated structures, cyanation attempts of 5-(chloromethyl)-3-phenyl-1,2,4-oxadiazoles **1** with excess KCN at room temperature in CH_3_CN have been investigated leading to trisubstituted acetonitrile **3** instead of anticipated product **2**. This result is in accord with a previous report where only one example (**3a**) has been exploited by providing very limited structural data [19]. Interestingly, increasing the reaction temperature to 100 °C yielded 1,2,4-oxadiazole-substituted propanes **4** as the major products which can only be interpreted via a decyanation pathway of cyanated oxadiazoles **3** (Figure 2).

Up to date, various methods have been reported for the conversion of organic nitriles into the parent alkanes; such as oxidative decyanation [20], dehydrocyanation [21] and more commonly, reductive decyanation [22]. Among them, the reductive decyanation is a widely used method employing metal hydrides [23], electrolysis [24], transition metal complexes [25], and alkali metals in a variety of solvents [26]. Generally, they require mostly an inert atmosphere as well as harsh reaction conditions. These facts constitute challenges to the development of mild reaction conditions in the reduction of nitrilated compounds.

In the light of the above considerations, we report here an unfamiliar example of non-reductive decyanation through the reaction of 5-(chloromethyl)-3-(substituted-phenyl)-1,2,4-oxadiazoles **1** with 2 equiv of KCN at 100 °C in a single step transformation.

## Results and Discussion

In the first part of this work, 5-(chloromethyl)-3-(substituted-phenyl)-1,2,4-oxadiazoles **1** were obtained via a previously published literature procedure by reacting *p*-substituted benzamidoximes with chloroacetyl chloride in refluxing benzene [27]. After having been obtained the starting oxadiazoles, our goal was to replace the chlorine atom with a CN^−^ anion by a simple S_N_2 reaction. For this purpose, 5-chloromethyl-1,2,4-oxadiazole **1a** was reacted with 10 equiv of KCN in CH_3_CN at reflux, after completion of the reaction on the basis of TLC, surprisingly we obtained a mixture of products **4** (major) and **3** (minor, Table 1, entry 1). This unexpected product distribution of the reaction prompted us to investigate the reaction of 5-(chloromethyl)-3-(*p*-substituted-phenyl)-1,2,4-oxadiazoles **1** with KCN.

Our initial effort was to investigate the limitation of the reaction. For this, the effect of temperature on this reaction was examined in CH_3_CN with various equivalents of KCN and varying reaction times (Table 1, entries 2–6). Treatment of **1a** with 10 equiv of KCN at 100 °C gave mainly product **4a** with a yield of 75%, as well as product **3a** in trace amounts even when heating at 110 °C. Due to the toxic nature of KCN, the minimum amount of KCN was determined for optimal formation of **4a**. When 2 equiv of KCN was used instead of 10 equiv at 100 °C in CH_3_CN, the reaction proceeded to give compound **4a** with a yield of 75% again, but 24 h for completion of the reaction were needed.

**Table 1:** Optimisation of reaction conditions.

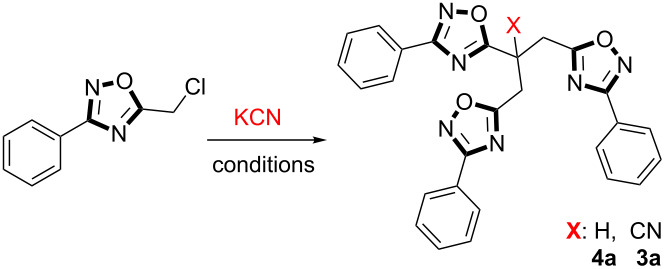				

Entry	KCN (equiv)^a^	Conditions	Yield (%)^b^	
			**4a**	**3a**

1	10	CH_3_CN, 80 °C, 3 h	55	32
2	10	CH_3_CN, 80 °C, 12 h	70	15
3	10	CH_3_CN, 100 °C, 12 h	75	trace
4	10	CH_3_CN, 110 °C, 12 h	75	trace
**5^c^**	**2**	**CH_3_CN, 100 °C, 24 h**	**75**	**trace**
6	10	CH_3_CN, rt, 12 h	trace	85
7	6	CH_3_CN, rt, 20 h	trace	85
**8^d^**	**4**	**CH_3_CN, rt, 24 h**	**trace**	**85**
9	2	CH_3_CN, rt, 24 h	–	52

^a^Equivalent of KCN respect to **1a. **^b^All yields were calculated after purification by flash chromatography. ^c^The most effective method for the formation of **4a. **^d^The most effective method for the formation of **3a**.

In further trials, we aimed to decrease the reaction temperature from 100 °C to room temperature in CH_3_CN using 10 equiv of KCN. In this case, the reaction ended with the formation of compound **3a** as a major product (85%) and decyanated product **4a** was obtained in trace amounts. To optimize the reaction conditions for compound **3a**, different equivalents of KCN have been tried. We were pleased to find that the use of 4 equiv KCN in CH_3_CN gave the desired product **3a** in high yield with a trace amount of decyanated product **4a**.

After having the optimized reaction conditions, 5-(chloromethyl)-3-(*p*-substituted-phenyl)-1,2,4-oxadiazoles **1** were reacted sequentially with 2 equiv of KCN in CH_3_CN at 100 °C and 4 equiv of KCN in CH_3_CN at room temperature giving the title compounds **3** and **4** as major products in high yields (75–85%, Table 2). Interestingly, incorporation of electron-poor substituents in compounds **1f** and **1g** with 2 equiv of KCN at 100 °C (method A) afforded only the products **4f** and **4g**, respectively, in a shorter reaction time (Table 2, entries 6 and 7).

**Table 2:** Formation of **3** and their parent alkanes **4**.

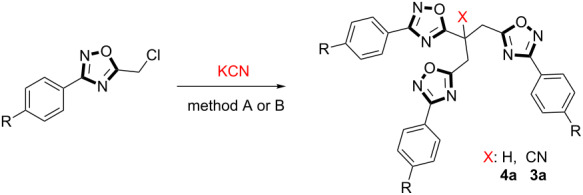							

Entry	Substrate	**4**	**3**	Yield (%)			
				Method A^a^		Method B^b^	
				**3**	**4**	**3**	**4**

**1**	**1a**: R = H	**4a**: R = H; X = H	**3a**: R = H; X = CN	trace	75	85	5
**2**	**1b**: R = Cl	**4b**: R = Cl; X = H	**3b**: R = Cl; X = CN	7	70	78	trace
**3**	**1c**: R = I	**4c**: R = I; X = H	**3c**: R = I; X = CN	5	68	72	trace
**4**	**1d**: R = F	**4d**: R = F; X = H	**3d**: R = F; X = CN	7	65	70	trace
**5**	**1e**: R = CH_3_	**4e**: R = CH_3_; X = H	**3e**: R = CH_3_; X = CN	trace	72	82	5
**6**	**1f**: R = NO_2_	**4f**: R = NO_2_; X = H	**3f**: R = NO_2_; X = CN	–	82^c^	78	15
**7**	**1g**: R = CF_3_	**4g**: R = CF_3_; X = H	**3g**: R = CF_3_; X = CN	–	78^d^	75	12
**8**	**1h**: R = OCH_3_	**4h**: R = OCH_3_; X = H	**3h**: R = OCH_3_; X = CN	5	70	78	trace
**9**	**1j**: R = SCH_3_	**4j**: R = SCH_3_; X = H	**3j**: R = SCH_3_; X = CN	7	72	76	trace

^a^CH_3_CN, 2 equiv KCN, 100 °C, 12 h ^b^CH_3_CN, 4 equiv KCN, rt, 24 h ^c^CH_3_CN, 2 equiv KCN, 100 °C, 6 h ^d^CH_3_CN, 2 equiv KCN, 100 °C, 8 h.

**Figure 3:**
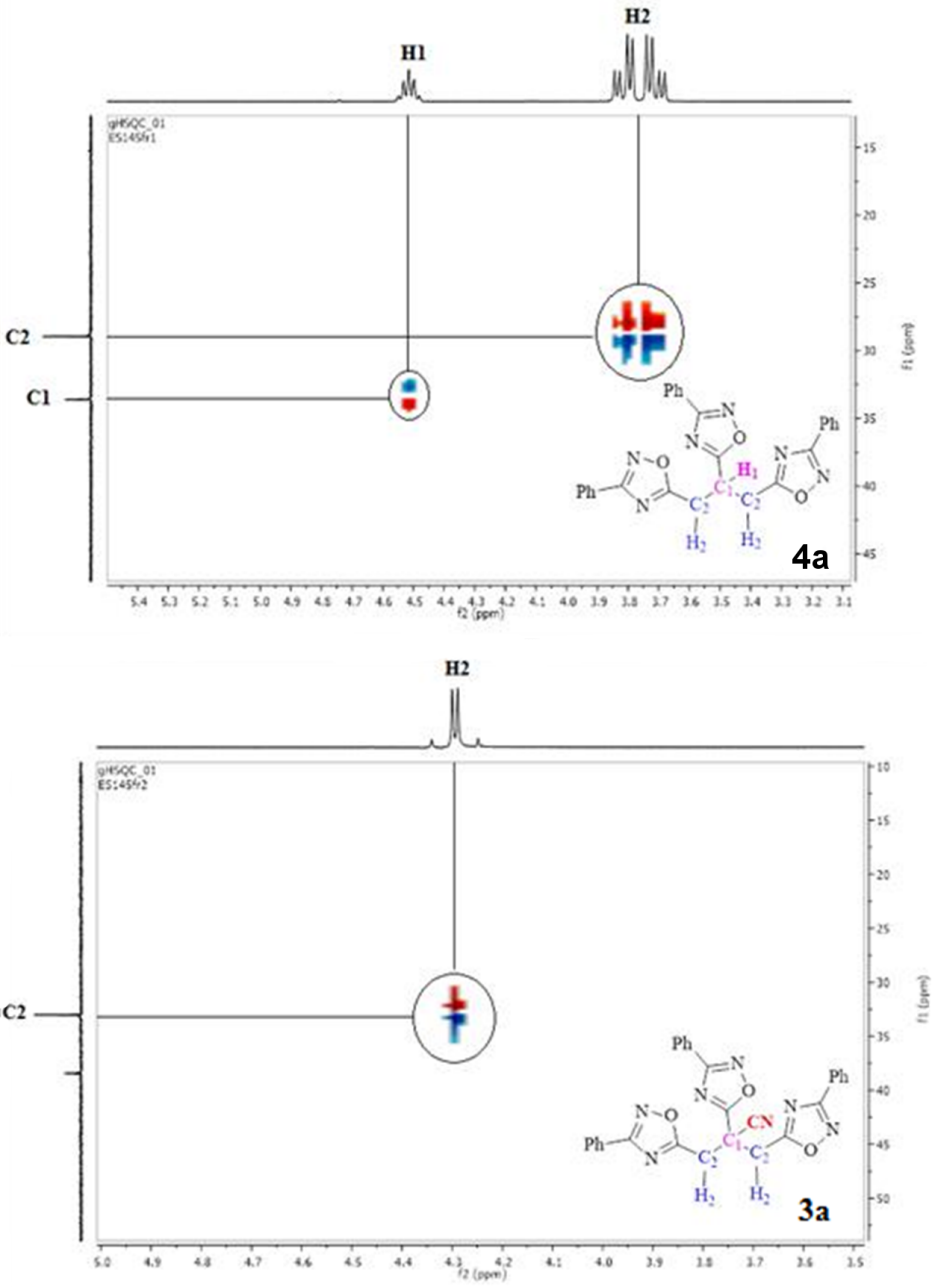
Expanded HSQC spectrum of **4a** and **3a**.

However, due to the replacement of the nitrile group with hydrogen after decyanation in product **4**, the methylenic protons and the methinic proton resonates at around 4–5 ppm as doublets and 5 ppm as pentet, respectively (Figure 3). The aliphatic protons of title compounds **3** and **4** were also assigned on the basis of HSQC and HMBC NMR experiments. The expanded HSQC spectrum of **3a** showed the C2 carbon at 35 ppm which corresponds to the methylenic hydrogens H2. In addition, the HSQC spectrum of **4a** showed that the H1 proton located between the methylene coupled with the C1 carbon and that the methylene protons H2 were incorporated in the C2 carbon signal.

**Figure 4:**
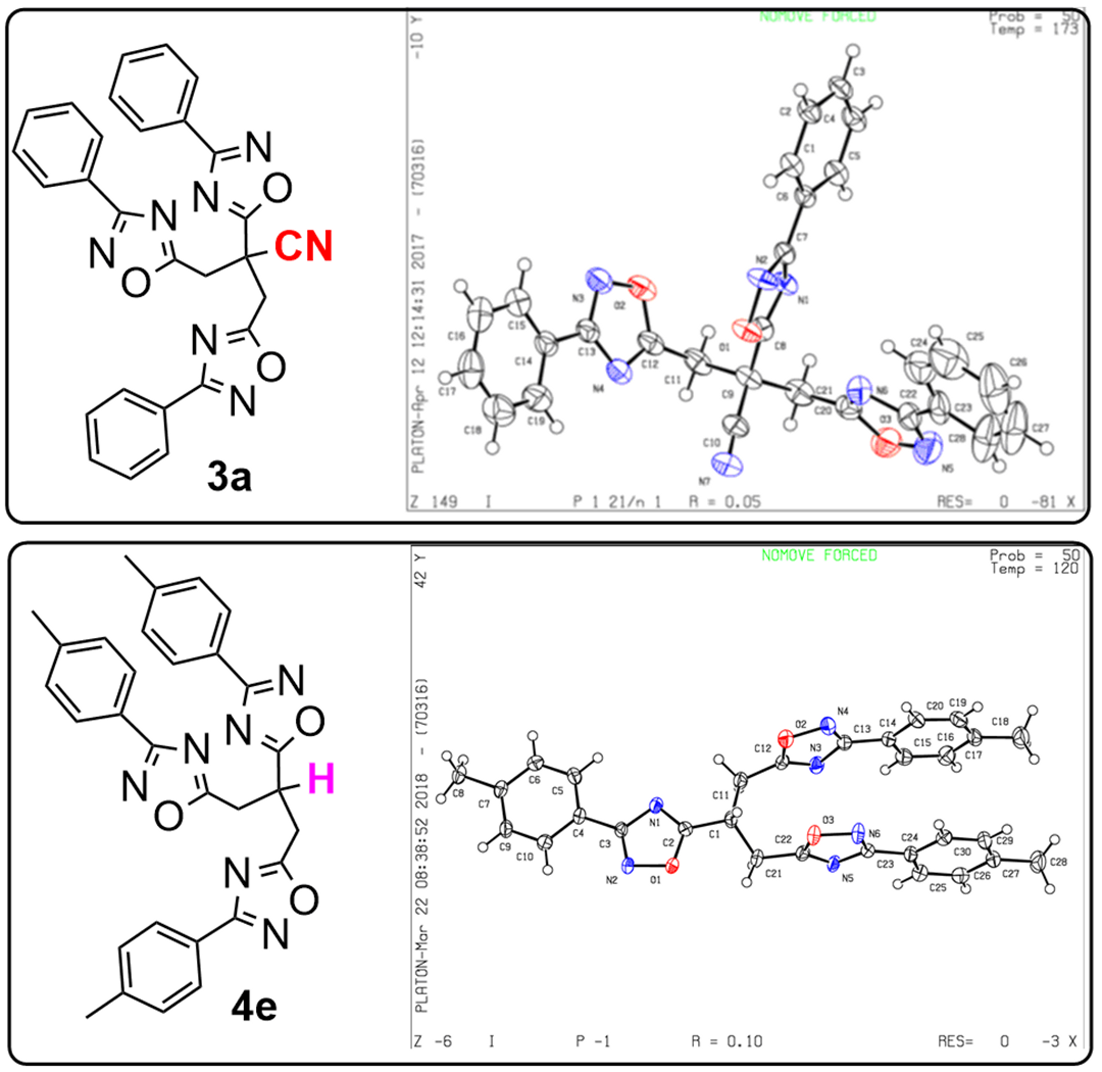
X-ray ORTEP plots of **3a** and **4e**.

Fortunately, the exact structures of compounds **3a** and **4e** were also confirmed by X-ray structures (Figure 4). The CIF data of **3a** and **4e** have been deposited at Cambridge Crystallographic Data Centre with the deposition numbers 1844118 and 1832133.

**Scheme 1:**
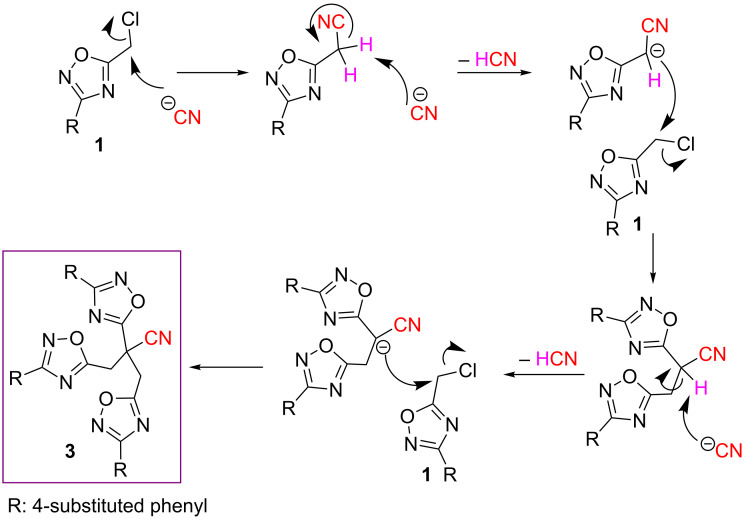
Plausible mechanism for the formation of **3**.

We propose a plausible mechanism for the transformation of chloromethyloxadiazoles **1** into the title products **3** and **4**. Accordingly, the reaction of **1** with a CN^−^ anion gives cyanomethyloxadiazole by simple S_N_2 reaction. Then, the acidic hydrogen adjacent to the nitrile group in the intermediate product is sequentially abstracted by a CN^−^ anion with the extrusion of an HCN molecule and a carbanion alpha to the nitrile group bearing a 1,2,4-oxadiazole ring is formed. The resulted carbanion undergoes a substitution reaction with another molecule of **1**, affording di-1,2,4-oxadiazole-substituted acetonitrile, which then undergoes a second substitution reaction with another molecule of **1**, in the same manner, finally affording title compound **3** (Scheme 1).

**Scheme 2:**
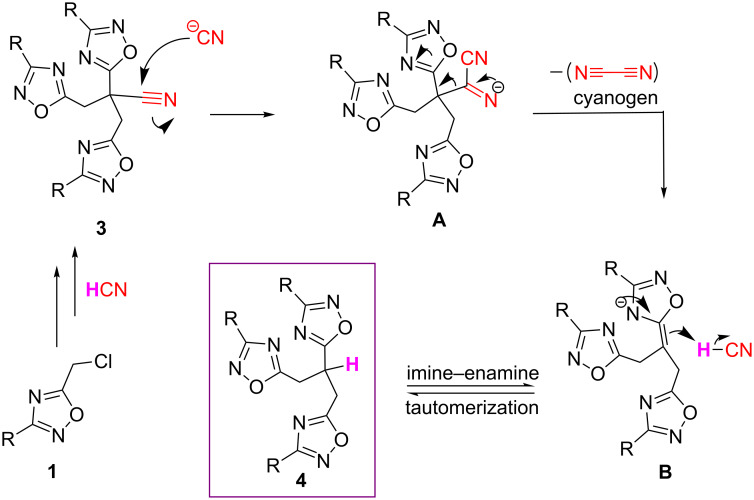
Plausible mechanism for the formation of **4** via decyanation of **3**.

Considering the formation of product **4**, as we believe, the temperature plays a significant role upon formation of it and in situ-formed HCN facilitates the decyanation (nitrile–alkane conversion) possibly since it is the only proton source in the reaction medium. This unfamiliar decyanation reaction presumably proceeds through product **3**. First, in the presence of CN^−^ anions, cyanoimine salt intermediate **A** forms by addition to a nitrile. Then, a possible cyanogen release would occur to form intermediate **B**. The imine–enamine tautomerization of **B** by the effect of in situ-generated HCN results in the desired decyanated product **4** (Scheme 2).

## Conclusion

In summary, we reported a convenient one-pot protocol for the synthesis of **3** and its transformation into **4** via a decyanation pathway by the reaction of 5-(chloromethyl)-3-(*p*-substituted-phenyl)-1,2,4-oxadiazoles **1** with KCN at different temperatures. This decyanation method is the first example for the conversion of a nitrile group into an alkane by using KCN–HCN associates through a possible release of cyanogen which is not detected or isolated. This synthetic protocol seems to be applicable for further decyanation processes containing a 1,2,4-oxadiazole moiety.

## Supporting Information

File 1 Experimental details, characterization data and copies of NMR spectra.
