# Supplementary material for: Reactions of 3-(p-substituted-phenyl)-5-chloromethyl-1,2,4-oxadiazoles with KCN leading to acetonitriles and alkanes via a non-reductive decyanation pathway
Source: Beilstein J Org Chem. 2018 Dec 10;14:3011–7. doi: 10.3762/bjoc.14.280 (PMC6296410; doi:10.3762/bjoc.14.280)
Supplement: File 1 — Experimental details, characterization data and copies of NMR spectra. [file Beilstein_J_Org_Chem-14-3011-s001.pdf]

BEILSTEIN JOURNAL OF ORGANIC CHEMISTRY

Supporting Information

for

Reactions of 3-(*p*-substituted-phenyl)-5-chloromethyl-1,2,4-oxadiazoles with KCN leading to acetonitriles and alkanes via a non-reductive decyanation pathway

Akın Sağırlı and Yaşar Dürüst

Beilstein J. Org. Chem. **2018,** *14,* 3011–3017. doi:10.3762/bjoc.14.280

Experimental details, characterization data and copies of NMR spectra

License and Terms: This is a supporting information file under the terms of the Creative Commons Attribution License (http://creativecommons.org/licenses/by/4.0). Please note that the reuse, redistribution and reproduction in particular requires that the authors and source are credited.
The license is subject to the *Beilstein Journal of Organic Chemistry* terms and conditions: (https://www.beilstein-journals.org/bjoc)

Table of contents

1.General information S2

2.General procedure for the synthesis of trisubstituted 1,2,4-oxadiazole-acetonitriles 3...........S2

3.General procedure for the synthesis of 1,2,3-trisubstituted 1,2,4-oxadiazole propanes 4 ....S6

4.^{1}H and ^{13}C NMR spectra of 3a–j S10

5.^{1}H and ^{13}C NMR spectra of 4a–j S19

6.HSQC and HMBC spectra of 3a... S28

7.HSQC and HMBC spectra of 4a... S29

8.References S30

1. General information

In order to dispose KCN waste properly, all KCN solutions were detoxified with hydrogen peroxide solution. 5-(Chloromethyl)-3-(substituted-phenyl)-1,2,4-oxadiazoles derivatives **1a–j** were synthesized prior to use following literature procedure [1]. ^{1}H and ^{13}C NMR (400 or 300 MHz for proton and 100 or 75 MHz for carbon, respectively) spectra were recorded in $CDCl_3$ at ambient temperature. LC–MS spectra were obtained from Waters 2695 Alliance Micromass ZQ instrument. High-resolution mass spectra (HRMS) of compounds were obtained on an orthogonal acceleration-TOF mass spectrometer and an FTMS (4.7 T) mass spectrometer. Single crystal X-ray diffraction data were obtained by Bruker Smart Apex II Quazar and Nonius Kappa CCD instruments. Melting points were determined with a Meltemp apparatus without corrections. All chemical shifts are reported in ppm relative to TMS. Coupling constants (*J*) are reported in Hz. Routine TLC analyses were carried out on pre-coated silica gel plates with fluorescent indicator. Flash column chromatography was performed on silica gel (230–400 Mesh ASTM). Stain solutions of potassium permanganate and iodine were used for visualization of the TLC spots.

2. General procedure for the synthesis of trisubstituted 1,2,4-oxadiazole-acetonitriles 3

Method B. A mixture of 5-(chloromethyl)-3-substitutedphenyl-1,2,4-oxadiazoles derivatives **1a–j** (0.75 mmol) and KCN (3 mmol, 195 mg) were stirred in CH_3CN (20 ml) at rt for 24 h. The reaction progress was followed by TLC and upon completion, the reaction mixture was concentrated in vacuo. The resulting residue was extracted with CH_2Cl_2 (25 ml), dried over Na_2SO_4, and the solvent was removed. Finally, crude products were purified by flash column chromatography on silica gel to afford the title compound **3** in a pure state.

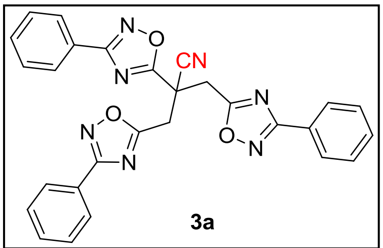

3a

2,3-Bis(3-phenyl-1,2,4-oxadiazol-5-yl)-2-((3-phenyl-1,2,4-oxadiazol-5-yl)methyl)propanenitrile (**3a**) Compound **3a** was prepared following method B using **1a** (0.75 mmol, 145 mg) and KCN (3 mmol, 195 mg) and stirring at rt for 24 h. Column chromatography yielded (106 mg, 85%) as a white solid. mp 125-127 ^{o}C. IR (KBr): ν = 3010, 2920, 2857, 2163 (weak-CN), 1595, 1570, 1526, 1445, 1361, 1302,1221,892 777, 704, 688 cm^{-1}. ^{1}H NMR (400 MHz, $CDCl_3$) δ 8.08 – 7.97 (m, 6H), 7.56 – 7.40 (m, 9H), 4.28 (d, *J* = 4.0 Hz, 4H). ^{13}C NMR (101 MHz, $CDCl_3$) δ 172.99, 172.26, 169.11, 168.57, 131.93, 131.61, 128.99, 128.90, 127.64, 127.54, 125.79, 125.39, 114.91(-CN), 38.42, 33.00. HRMS (-APCl-TOF) calcd for $C_{28}H_{18}N_7O_3$ $[M-H]^+$ 500.1471, found 500.1487.

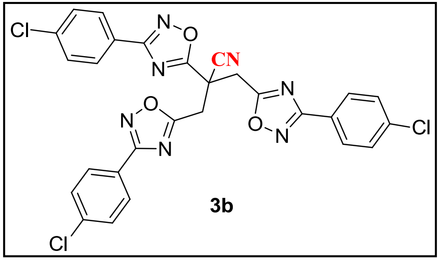

3b

2,3-Bis(3-(4-chlorophenyl)-1,2,4-oxadiazol-5-yl)-2-((3-(4-chlorophenyl)-1,2,4-oxadiazol-5-yl)methyl)propanenitrile (**3b**) Compound **3b** was prepared following method B using **1b** (0.75 mmol, 172 mg) and KCN (3 mmol, 195 mg) and stirring at rt for 24 h. Column chromatography yielded (118 mg, 78%) as a light yellow solid. mp 156-158 °C. IR (KBr): ν = 3008, 2925, 2860, 2161 (weak-CN), 1587, 1562, 1471, 1407, 1344, 1183, 1092, 1012, 902, 832, 733 cm^{-1} ^{1}H NMR (400 MHz, $CDCl_3$) δ 7.99 (d, *J* = 8.6 Hz, 2H), 7.94 (d, *J* = 8.6 Hz, 4H), 7.47 (d, *J* = 8.6 Hz, 2H), 7.44 (d, *J* = 8.6 Hz, 4H), 4.26 (d, *J* = 3.6 Hz, 4H). ^{13}C NMR (101 MHz, $CDCl_3$) δ 173.14, 172.38, 168.36, 167.82, 138.36, 137.94, 129.42, 129.30, 128.90, 128.81, 124.18, 123.75, 114.70(-CN), 38.45, 33.13. HRMS (+APCI-TOF) calcd for $C_{28}H_{17}Cl_3N_7O_3$ $[M+H]^+$ 604.0458, found 604.0489.

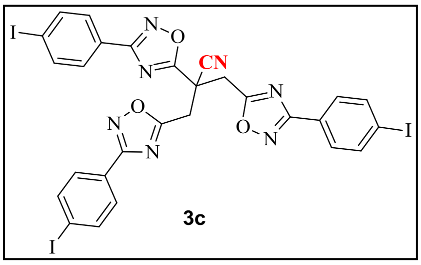

3c

2,3-Bis(3-(4-iodophenyl)-1,2,4-oxadiazol-5-yl)-2-((3-(4-iodophenyl)-1,2,4-oxadiazol-5-yl)methyl)propanenitrile (**3c**) Compound **3c** was prepared following method B using **1c** (0.75 mmol, 240 mg) and KCN (3 mmol, 195 mg) and stirring at rt for 24 h. Column chromatography yielded (158 mg, 72%) as a light yellow solid. mp 220-222 °C. IR (KBr): ν = 3010, 2920, 2852, 2160 (weak-CN), 1584, 1557, 1465, 1397, 1354, 1275, 1004, 911, 826, 746, 728 cm^{-1} ^{1}H NMR (400 MHz, $CDCl_3$) δ 7.81 (dd, *J* = 15.3, 8.2 Hz, 6H), 7.75 (d, *J* = 8.3 Hz, 2H), 7.70 (d, *J* = 8.3 Hz, 4H), 4.23 (d, *J* = 3.9 Hz, 4H). ^{13}C NMR (101 MHz, $CDCl_3$) δ 172.37, 168.07, 159.15, 156.46, 142.19, 138.34, 138.22, 128.98, 128.93, 125.17, 124.73, 114.67(-CN), 98.55, 38.43, 33.12. HRMS (-APCI-TOF) calcd for $C_{28}H_{15}I_3N_7O_3$ $[M-H]^+$ 877.8370, found 877.8362.

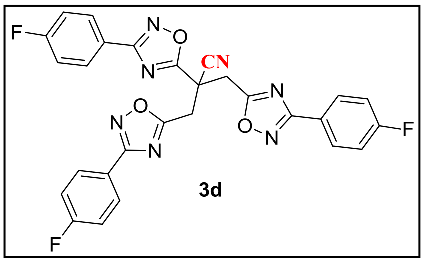

3d

2,3-Bis(3-(4-fluorophenyl)-1,2,4-oxadiazol-5-yl)-2-((3-(4-fluorophenyl)-1,2,4-oxadiazol-5-yl)methyl)propanenitrile (**3d**) Compound **3d** was prepared following method B using **1d** (0,75 mmol, 159 mg) and KCN (3 mmol, 195 mg) and stirring at rt for 24 h. Column chromatography yielded (97 mg, 70%) as a white solid. mp 139-141 °C. IR (KBr): ν = 3010, 2922, 2851, 2162 (weak-CN), 1606, 1573, 1482, 1416, 1354, 1219, 1155, 843, 759, 747, 601 cm^{-1} ^{1}H NMR (300 MHz, $CDCl_3$) δ 8.10 – 7.97 (m, 6H), 7.22 – 7.11 (m, 6H), 4.25 (d, *J* = 1.4 Hz, 4H). ^{13}C NMR (75 MHz, $CDCl_3$) δ 173.31, 172.54, 168.54, 167.99, 130.19, 130.04, 129.92, 122.16, 116.73, 116.59, 116.43, 116.29, 115.02(-CN), 38.67, 33.34. HRMS (-ESI-TOF) calcd for $C_{28}H_{15}F_3N_7O_3$ $[M-H]^+$ 554.1188, found 554.1206.

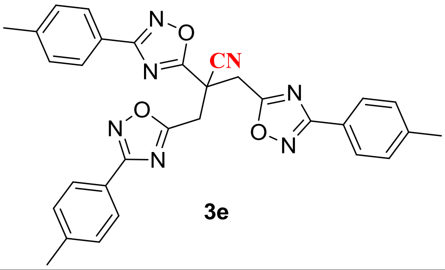

3e

2,3-Bis(3-(*p*-tolyl)-1,2,4-oxadiazol-5-yl)-2-((3-(*p*-tolyl)-1,2,4-oxadiazol-5-yl)methyl)propanenitrile (**3e**) Compound **3e** was prepared following method B using **1e** (0.75 mmol, 156 mg) and KCN (3 mmol, 195 mg) and stirring at rt for 24 h. Column chromatography yielded (111 mg, 82%) as a white solid. mp 131-133 °C. IR (KBr): ν = 3002, 2922, 2856, 2161 (weak-CN), 1592, 1570, 1478, 1411, 1363, 1219, 1113, 889, 822, 744 cm^{-1} ^{1}H NMR (400 MHz, $CDCl_3$) δ 7.89 (dd, *J* = 14.9, 7.7 Hz, 6H), 7.25 (t, *J* = 10.2 Hz, 6H), 4.25 (d, *J* = 3.9 Hz, 4H), 2.39 (d, *J* = 6.7 Hz, 9H). ^{13}C NMR (101 MHz, $CDCl_3$) δ 172.93, 172.26, 169.19, 168.65, 142.52, 142.13, 129.94, 129.48, 127.46, 123.07, 122.68, 115.14(-CN), 38.48, 33.03, 21.72. LC—MS (70 eV): (m/z, %)= 542.8 (100) $[M-H]^+$. HRMS (-APCl-TOF) calcd for $C_{31}H_{25}N_7O_3$ $[M-H]^+$ 542.1941, found 542.1920.

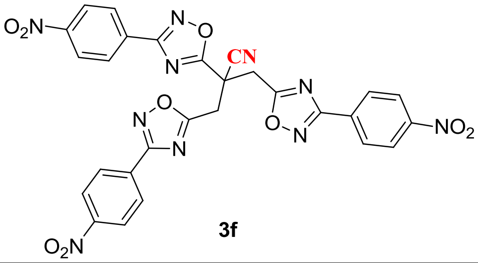

3f

2,3-Bis(3-(4-nitrophenyl)-1,2,4-oxadiazol-5-yl)-2-((3-(4-nitrophenyl)-1,2,4-oxadiazol-5-yl)methyl)propanenitrile (**3f**) Compound **3f** was prepared following method B using **1f** (0.75 mmol, 179 mg) and KCN (3 mmol, 195 mg) and stirring at rt for 24 h. Column chromatography yielded (124 mg, 78%) as a light yellow solid. mp184-186 °C. IR (KBr): ν = 3010, 2920, 2852, 2158 (weak-CN), 1640, 1618, 1519, 1418, 1341, 1108, 851, 720, 618 cm^{-1} ^{1}H NMR (400 MHz, $CDCl_3$) δ 7.83 (d, *J* = 8.2 Hz, 2H), 7.79 (d, *J* = 8.2 Hz, 4H), 7.75 (d, *J* = 8.3 Hz, 2H), 7.70 (d, *J* = 8.3 Hz, 4H), 4.23 (d, *J* = 3.9 Hz, 4H). ^{13}C NMR (75 MHz, $CDCl_3$) δ 174.43, 173.73, 167.57, 166.97, 149.86, 149.64, 131.69, 131.18, 128.73, 128.56, 124.31, 124.21, 114.85(-CN), 39.17, 33.89. HRMS (-APCl-TOF) calcd for $C_{28}H_{16}N_{10}O_9$ $[M-H]^+$ 635.1023, found 635.1041.

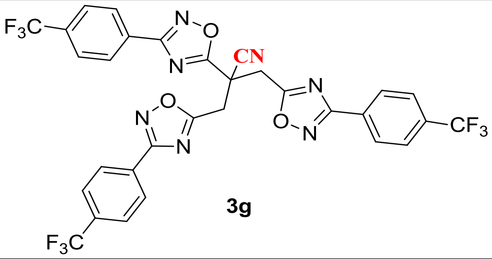

3g

2,3-Bis(3-(4-(trifluoromethyl)phenyl)-1,2,4-oxadiazol-5-yl)-2-((3-(4-(trifluoromethyl)phenyl)-1,2,4-oxadiazol-5-yl)methyl)propanenitrile (**3g**) Compound **3g** was prepared following method B using **1g** (0,75 mmol, 197 mg) and KCN (3 mmol, 195 mg) and stirring at rt for 24h. Column chromatography yielded (132 mg, 75%) as a white solid. mp 199-201 °C. IR (KBr): ν = 3005, 2925, 2852, 2160 (weak-CN), 1590, 1570, 1541, 1416, 1320, 1161, 1119, 1064, 849, 758, 705 cm^{-1} ^{1}H NMR (400 MHz, $CDCl_3$) δ 8.19 (d, *J* = 8.2 Hz, 2H), 8.13 (d, *J* = 8.2 Hz, 4H), 7.76 (d, *J* = 8.6 Hz, 2H), 7.73 (d, *J* = 8.2 Hz, 4H), 4.30 (d, *J* = 3.4 Hz, 4H). ^{13}C NMR

(101 MHz, $CDCl_3$) δ 173.46, 172.63, 168.19, 167.64, 133.62, 133.30, 129.00, 128.57, 128.00, 127.88, 124.91, 122.21, 114.51(-CN), 38.53, 33.31. HRMS (+APCl-TOF) calcd for $C_{31}H_{17}F_9N_7O_3$ $[M+H]^+$ 706.1249, found 706.1245.

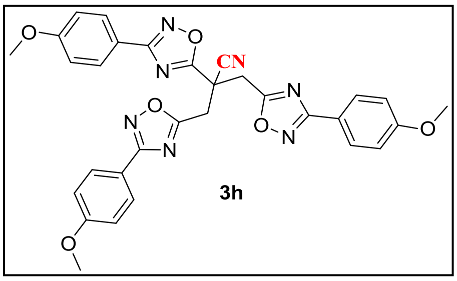

3h

2,3-Bis(3-(4-methoxyphenyl)-1,2,4-oxadiazol-5-yl)-2-((3-(4-methoxyphenyl)-1,2,4-oxadiazol-5-yl)methyl)propanenitrile (**3h**) Compound **3h** was prepared following method B using **1h** (0,75 mmol, 168 mg) and KCN (3 mmol, 195 mg) and stirring at rt for 24 h. Column chromatography yielded (115 mg, 78%) as a brown solid. mp 140-142 °C. IR (KBr): ν = 3002, 2933, 2844, 2161 (weak-CN), 1610, 1594, 1570, 1479, 1421, 1251, 1171, 1028, 834, 752, 614 cm^{-1} ^1H NMR (400 MHz, $CDCl_3$) δ 7.97 (d, *J* = 8.6 Hz, 2H), 7.93 (d, *J* = 8.6 Hz, 4H), 6.95 (dd, *J* = 10.6, 8.9 Hz, 6H), 4.23 (d, *J* = 3.2 Hz, 4H), 3.85 (s, 3H), 3.84 (s, 6H). ^{13}C NMR (101 MHz, $CDCl_3$) δ 172.82, 172.13, 168.87, 168.33, 162.55, 162.29, 129.44, 129.31, 118.33, 117.90, 115.19(-CN), 114.48, 114.39, 55.53, 55.49, 38.48, 33.04. LC—MS (70 eV): (m/z, %)= 592.4 (100) $[M+H]^+$. HRMS (-APCl-TOF) calcd for $C_{31}H_{24}N_7O_6$ $[M-H]^+$ 590.1788, found 590.1739.

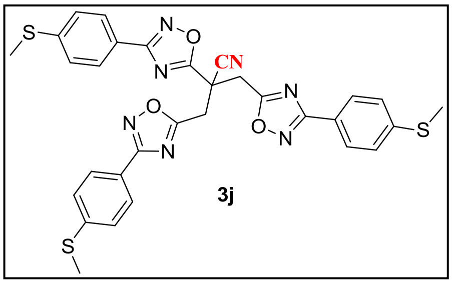

3j

2,3-Bis(3-(4-(methylthio)phenyl)-1,2,4-oxadiazol-5-yl)-2-((3-(4-(methylthio)phenyl)-1,2,4-oxadiazol-5-yl)methyl)propanenitrile (**3j**) Compound **3j** was prepared following method B using **1j** (0,75 mmol, 180 mg) and KCN (3 mmol, 195 mg) and stirring at rt for 24 h. Column chromatography yielded (121 mg, 76%) as a brown solid. mp 160-162 °C. IR (KBr): ν = 3000, 2926, 2856, 2161 (weak-CN), 1590, 1556, 1474, 1407, 1360, 1182, 1120, 900, 834, 748, 502 cm^{-1} ^1H NMR (400 MHz, $CDCl_3$) δ 7.94 (d, *J* = 8.4 Hz, 2H), 7.90 (d, *J* = 8.7 Hz, 4H), 7.30 (d, *J* = 8.5 Hz, 2H), 7.27 (d, *J* = 8.5 Hz, 4H), 4.25 (d, *J* = 3.9 Hz, 4H), 2.52 (s, 3H), 2.51 (s, 6H). ^{13}C NMR (101 MHz, $CDCl_3$) δ 172.86, 172.15, 168.77, 168.23, 144.16, 143.62, 127.83, 127.75, 125.78, 121.98, 121.51, 114.93(-CN), 38.43, 33.04, 15.01. HRMS (+APCl-TOF) calcd for $C_{31}H_{26}N_7O_3S_3$ $[M+H]^+$ 640.1259, found 615.1281.

3. General procedure for the synthesis of 1,2,3-trisubstituted 1,2,4-oxadiazole propanes 4

Method A. A mixture of 5-(chloromethyl)-3-(substituted-phenyl)-1,2,4-oxadiazoles derivatives **1a-j** (0.75mmol) and KCN (1,50 mmol, 98mg) were heated in CH_3CN (20 ml) at 100 °C for 12 h except **4f** and **4g**. The reaction progress was followed by TLC and upon completion, the reaction mixture was concentrated in vacuo. The resulting residue was extracted with CH_2Cl_2 (25 ml), dried over Na_2SO_4, and the solvent was removed. Finally, crude products were purified by flash column chromatography on silica gel to afford the title compound **4** in a pure state.

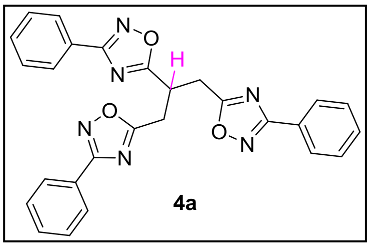

4a

5,5',5''-(Propane-1,2,3-triyl)tris(3-phenyl-1,2,4-oxadiazole) (**4a**) Compound **4a** was prepared following method A using **1a** (0.75 mmol, 145 mg) and KCN (1.50 mmol, 98 mg) and stirring at 100 °C for 12 h. Column chromatography yielded (89 mg, 75%) as a white solid. mp 98–100 °C. IR (KBr): ν = 2918, 2951, 1645, 1573, 1446, 1363, 1288, 1172, 1114, 1072, 1003, 898, 692 cm^{-1}. ^1H NMR (400 MHz, $CDCl_3$) δ 8.06 – 7.97 (m, 6H), 7.53 – 7.38 (m, 9H), 4.51 (p, *J* = 6.8 Hz, 1H), 3.81 (dd, *J* = 16.4, 6.6 Hz, 2H), 3.71 (dd, *J* = 16.5, 7.2 Hz, 2H). ^{13}C NMR (101 MHz, $CDCl_3$) δ 178.19, 175.76, 168.48, 168.39, 131.38, 131.30, 128.84, 128.81, 127.50, 127.44, 126.28, 126.27, 33.61, 28.94. HRMS (-APCl-TOF) calcd for $C_{27}H_{19}N_6O_3$ $[M-H]^+$ 475.1519, found 475.1556.

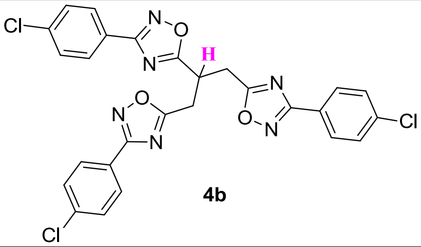

4b

5,5',5''-(Propane-1,2,3-triyl)tris(3-(4-chlorophenyl)-1,2,4-oxadiazole) (**4b**) Compound **4b** was prepared following method A using **1b** (0.75 mmol, 172 mg) and KCN (1.50 mmol, 98 mg) and stirring at 100 °C for 12 h. Column chromatography yielded (101 mg, 70%) as a yellow solid. mp 168-170 °C. IR (KBr): ν = 2918, 2847, 1588, 1561, 1472, 1409, 1365, 1091, 1014, 902, 836, 763 cm^{-1}. ^1H NMR (400 MHz, $CDCl_3$) δ 7.97 (d, *J* = 8.6 Hz, 2H), 7.93 (d, *J* = 8.6 Hz, 4H), 7.42 (d, *J* = 8.6 Hz, 6H), 4.50 (p, *J* = 6.9 Hz, 1H), 3.81 (dd, *J* = 16.4, 6.4 Hz, 2H), 3.70 (dd, *J* = 16.4, 7.2 Hz, 2H). ^{13}C NMR (101 MHz, $CDCl_3$) δ 178.29, 175.86, 167.74, 167.62, 137.70, 137.59, 129.23, 129.16, 128.78, 128.70, 124.71, 33.62, 29.66, 28.97. HRMS (+APCl-TOF) calcd for $C_{27}H_{18}Cl_3N_6O_3$ $[M+H]^+$ 579.0506, found 579.0535.

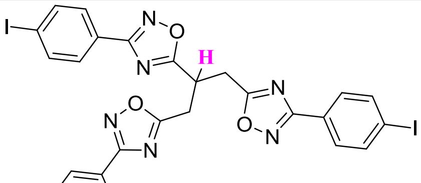
4c

5,5',5''-(Propane-1,2,3-triyl)tris(3-(4-iodophenyl)-1,2,4-oxadiazole) (**4c**) Compound **4c** was prepared following method A using **1c** (0,75 mmol, 240 mg) and KCN (1,50 mmol, 98 mg) and stirring at 100°C for 12 h. Column chromatography yielded (145 mg, 68%) as a white solid. mp 169-171 °C. IR (KBr): ν = 2930, 2820, 1593, 1570, 1418, 1321, 1169, 1130, 1065, 849, 766, 595 cm^{-1}. ^{1}H NMR (400 MHz, $CDCl_3$) δ 7.83 – 7.80 (m, 2H), 7.79 (d, *J* = 4.5 Hz, 2H), 7.75 (d, *J* = 6.9 Hz, 4H), 7.68 (d, *J* = 8.2 Hz, 4H), 4.48 (p, *J* = 6.8 Hz, 1H), 3.79 (dd, *J* = 16.4, 6.4 Hz, 2H), 3.67 (dd, *J* = 16.4, 7.4 Hz, 2H). ^{13}C NMR (101 MHz, $CDCl_3$) δ 178.38, 175.97, 168.07, 167.94, 138.27, 138.20, 129.04, 128.95, 125.73, 124.62, 98.42, 98.29, 33.67, 29.03. HRMS (-APCl-TOF) calcd for $C_{27}H_{16}I_3N_6O_3$ $[M-H]^+$ 852.8418, found 852.8392.

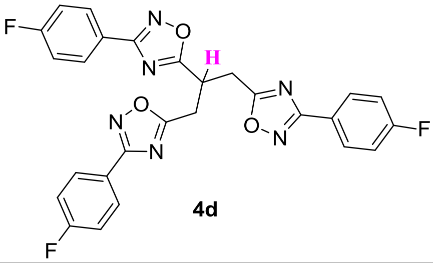
4d

5,5',5''-(Propane-1,2,3-triyl)tris(3-(4-fluorophenyl)-1,2,4-oxadiazole) (**4d**) Compound **4d** was prepared following method A using **1d** (0.75 mmol, 159 mg) and KCN (1.50 mmol, 98 mg) and stirring at 100 °C for 12 h. Column chromatography yielded (86 mg, 65%) as a yellow solid. mp 141-143 °C. IR (KBr): ν = 2928, 2815, 1605, 1573, 1481, 1416, 1356, 1226, 1158, 900, 842, 751 cm^{-1}. ^{1}H NMR (300 MHz, $CDCl_3$) δ 8.06 – 7.96 (m, 6H), 7.19 – 7.09 (m, 6H), 4.50 (p, *J* = 6.8 Hz, 1H), 3.81 (dd, *J* = 16.5, 6.5 Hz, 2H), 3.69 (dd, *J* = 16.4, 7.2 Hz, 2H). ^{13}C NMR (75 MHz, $CDCl_3$) δ 178.47, 176.04, 167.92, 167.80, 129.98, 129.89, 129.77, 122.65, 116.51, 116.44, 116.21, 116.15, 33.81, 29.18. HRMS (-APCl-TOF) calcd for $C_{27}H_{16}F_3N_6O_3$ $[M-H]^+$ 529.1236, found 529.1192.

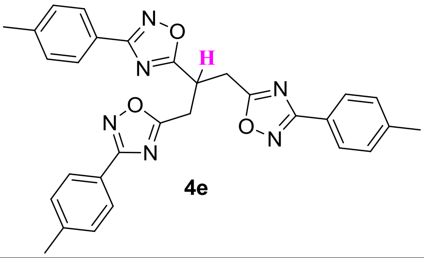
4e

5,5',5''-(Propane-1,2,3-triyl)tris(3-(*p*-tolyl)-1,2,4-oxadiazole) (**4e**) Compound **4e** was prepared following method A using **1e** (0.75 mmol, 156 mg) and KCN (1.50 mmol, 98 mg) and stirring at 100 °C for 12 h. Column chromatography yielded (93 mg, 72%) as a light yellow solid. mp 123-125 °C. IR (KBr): ν = 2918, 2851, 1593, 1567, 1480, 1411, 1349, 1080, 1013, 907, 824, 745 cm^{-1}. ^{1}H NMR (400 MHz, $CDCl_3$) δ 8.13 – 7.67 (m, 6H), 7.43 – 7.08 (m, 6H), 4.49 (p, *J* = 7.0 Hz, 1H), 3.79 (dd, *J* = 16.4, 6.5 Hz, 2H), 3.69 (dd, *J* = 16.4, 7.2 Hz, 2H), 2.39 (s, 9H). ^{13}C NMR (101 MHz, $CDCl_3$) δ 178.16, 175.75, 168.56, 168.48, 141.86,

141.76, 129.84, 129.42, 127.55, 127.42, 123.56, 33.56, 29.01, 21.66. HRMS (-APCl-TOF) calcd for $C_{30}H_{25}N_6O_3$ $[M-H]^+$ 517.1988, found 517.2024.

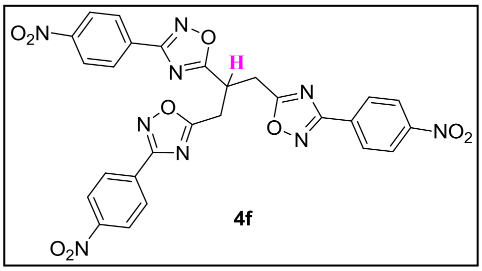

4f

5,5',5''-(Propane-1,2,3-triyl)tris(3-(4-nitrophenyl)-1,2,4-oxadiazole) (**4f**) Compound **4f** was prepared following method A using **1f** (0,75 mmol, 179 mg) and KCN (1.50 mmol, 98 mg) and stirring at 100 °C for 6 h. Column chromatography yielded (125 mg, 82%) as a light yellow solid. mp 169-171 °C. IR (KBr): ν = 2917, 2848, 1610, 1571, 1514, 1416, 1336, 1105, 907, 852, 749, 718 cm^{-1}. ^1H NMR (400 MHz, $CDCl_3$) δ 8.35 – 8.29 (m, 6H), 8.25 – 8.18 (m, 6H), 4.59 (p, *J* = 6.8 Hz, 1H), 3.90 (dd, *J* = 16.5, 6.7 Hz, 2H), 3.80 (dd, *J* = 16.5, 7.0 Hz, 2H). ^{13}C NMR (101 MHz, $CDCl_3$) δ 178.80, 176.40, 167.07, 166.96, 149.71, 149.65, 131.90, 131.83, 128.47, 128.39, 124.15, 124.11, 33.57, 28.97. HRMS (-APCl-TOF) calcd for $C_{27}H_{16}N_9O_9$ $[M-H]^+$ 610.1071, found 610.1097.

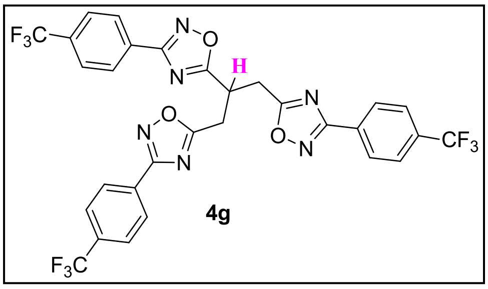

4g

5,5',5''-(Propane-1,2,3-triyl)tris(3-(4-(trifluoromethyl) phenyl)-1,2,4-oxadiazole) (**4g**) Compound **4g** was prepared following method A using **1g** (0,75 mmol, 197 mg) and KCN (1.50 mmol, 98 mg) and stirring at 100 °C for 8 h. Column chromatography yielded (133 mg, 78%) as a yellow solid. mp 149-151 °C. IR (KBr): ν = 2918, 2847, 1588, 1561, 1472, 1409, 1365, 1091, 1014, 902, 836, 763 cm^{-1}. ^1H NMR (400 MHz, $CDCl_3$) δ 8.17 (d, *J* = 8.1 Hz, 2H), 8.11 (d, *J* = 8.1 Hz, 4H), 7.74 (d, *J* = 8.3 Hz, 2H), 7.70 (d, *J* = 8.2 Hz, 4H), 4.57 (p, *J* = 6.9 Hz, 1H), 3.87 (dd, *J* = 16.5, 6.4 Hz, 2H), 3.75 (dd, *J* = 16.5, 7.3 Hz, 2H). ^{13}C NMR (101 MHz, $CDCl_3$) δ 178.53, 176.10, 167.56, 167.41, 133.31, 132.98, 129.50, 127.85, 127.73, 125.86, 124.97, 122.27, 33.65, 29.01. HRMS (+APCl-TOF) calcd for $C_{30}H_{18}F_9N_6O_3$ $[M+H]^+$ 681.1297, found 681.1294.

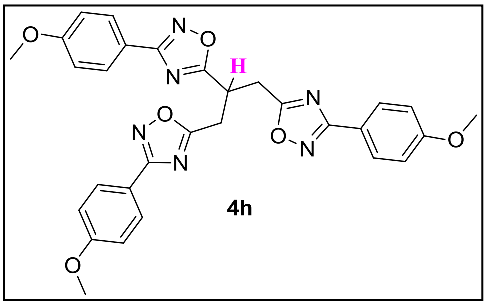

4h

5,5',5''-(Propane-1,2,3-triyl)tris(3-(4-methoxyphenyl)-1,2,4-oxadiazole) (**4h**) Compound **4h** was prepared following method A using **1h** (0,75 mmol, 168 mg) and KCN (1.50 mmol, 98 mg) and stirring at 100 °C for 12 h. Column chromatography yielded (99 mg, 70%) as a light yellow solid. mp 146-148 °C. IR (KBr): ν = 2924, 2852, 1610, 1588, 1566, 1479, 1424, 1357, 1253, 1172, 1023, 836, 751 cm^{-1}. ^1H NMR (400 MHz, $CDCl_3$) δ 8.00 –

7.91 (m, 6H), 6.95 (t, *J* = 8.5 Hz, 6H), 4.47 (p, *J* = 6.9 Hz, 1H), 3.85 (s, 9H), 3.78 (dd, *J* = 16.4, 6.5 Hz, 2H), 3.67 (dd, *J* = 16.4, 7.3 Hz, 2H). ^{13}C NMR (101 MHz, $CDCl_3$) δ 178.06, 175.63, 168.24, 168.16, 162.12, 162.05, 129.35, 129.24, 129.17, 118.83, 114.33, 114.29, 55.47, 33.68, 29.04. LC—MS (70 eV): (m/z, %)= 567.7 (100) $[M+H]^+$. HRMS (-APCl-TOF) calcd for $C_{30}H_{25}N_6O_6$ $[M-H]^+$ 565.1836, found 565.1884.

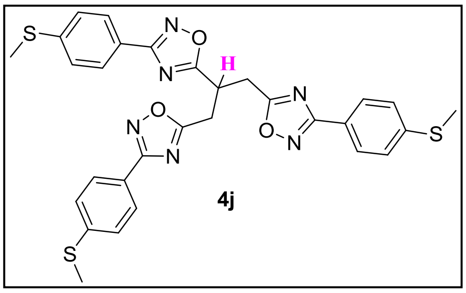

5,5',5''-(Propane-1,2,3-triyl)tris(3-(4-(methylthio)phenyl)-1,2,4-oxadiazole) (**4j**) Compound **4j** was prepared following method A using **1j** (0.75 mmol, 180 mg) and KCN (1.50 mmol, 98 mg) and stirring at 100 °C for 12 h. Column chromatography yielded (99 mg, 72%) as a light yellow solid. mp 111-113 °C. IR (KBr): ν = 2918, 2845, 1589, 1556, 1470, 1407, 1357, 1114, 1087, 904, 823, 745 cm^{-1}. ^{1}H NMR (400 MHz, $CDCl_3$) δ 7.94 (d, *J* = 10.1 Hz, 4H), 7.89 (d, *J* = 8.4 Hz, 4H), 7.30 – 7.25 (m, 4H), 4.49 (p, *J* = 6.7 Hz, 1H), 3.80 (dd, *J* = 16.4, 6.4 Hz, 2H), 3.68 (dd, *J* = 16.4, 7.4 Hz, 2H). ^{13}C NMR (101 MHz, $CDCl_3$) δ 178.07, 175.63, 168.15, 168.05, 143.28, 143.13, 127.78, 127.73, 127.66, 125.82, 125.76, 122.52, 33.64, 28.99, 15.06. HRMS (+APCl-TOF) calcd for $C_{30}H_{27}N_6O_3S_3$ $[M+H]^+$ 615.1307, found 615.1328.

^{1}H and ^{13}C NMR spectra of 3a–j

^{1}H NMR Spectrum of **3a**

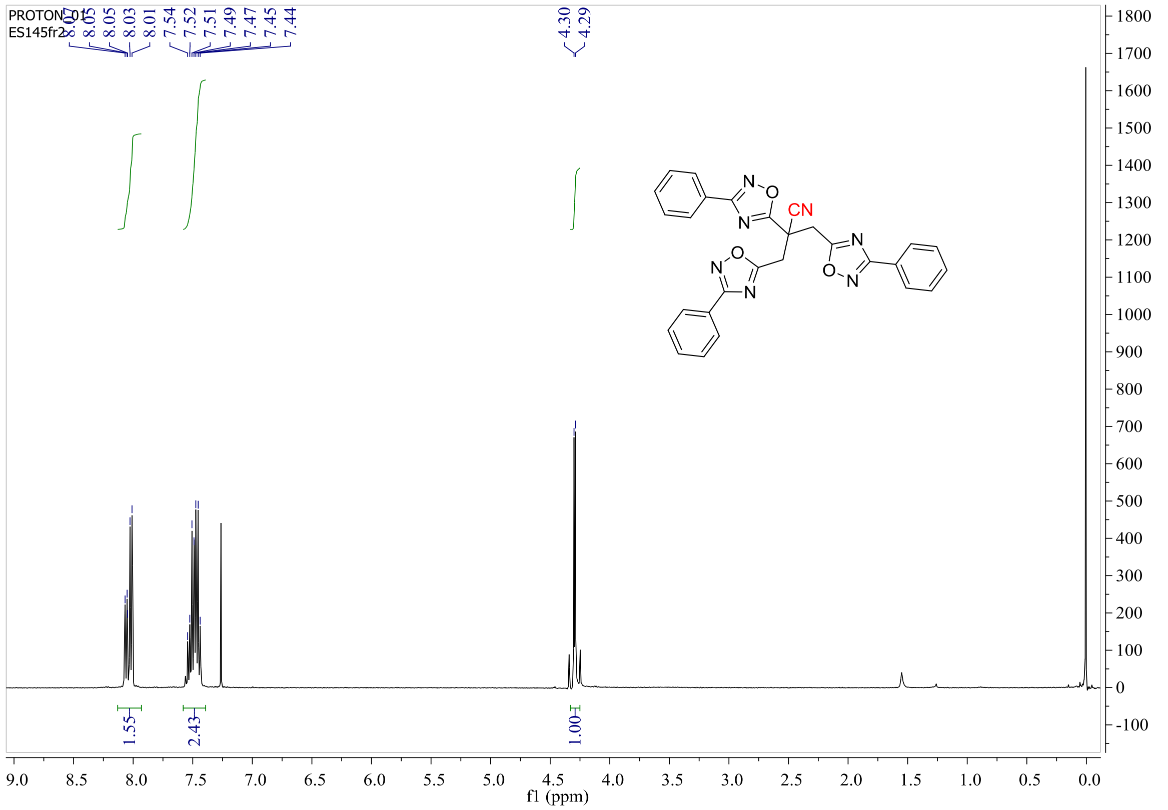

^{13}C NMR Spectrum of **3a**

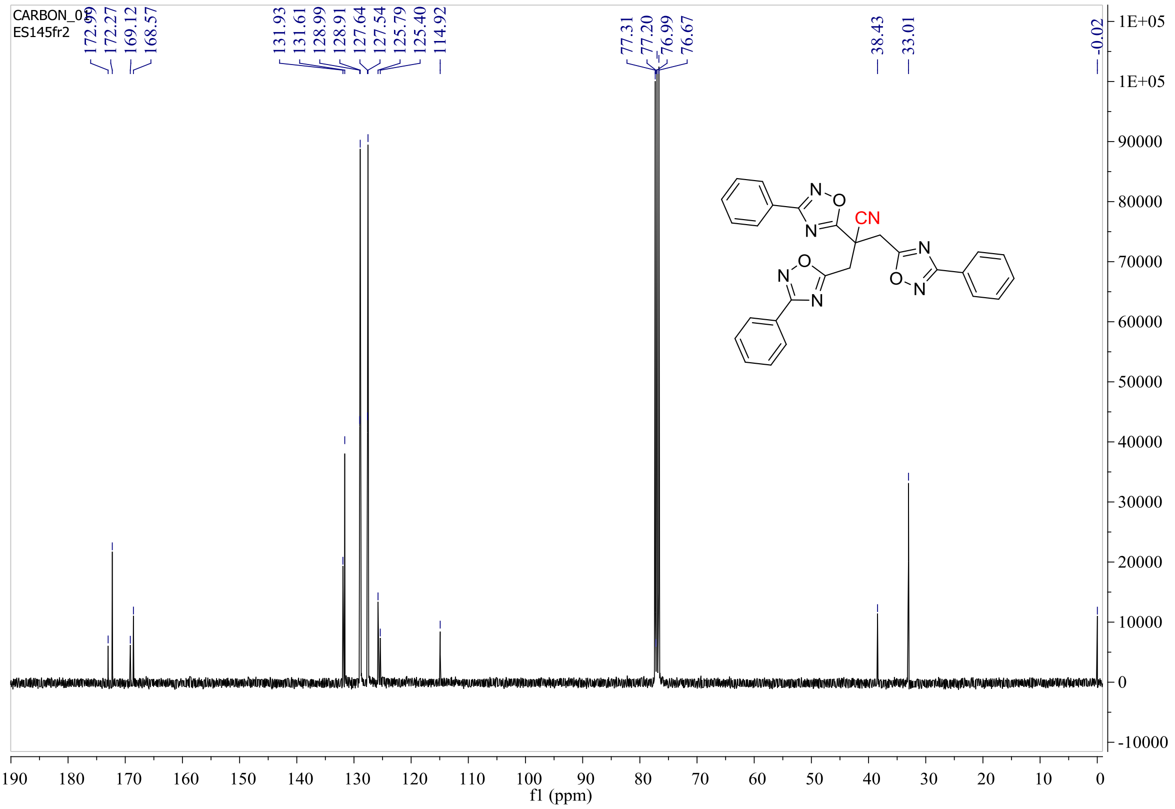

^{1}H NMR Spectrum of **3b**

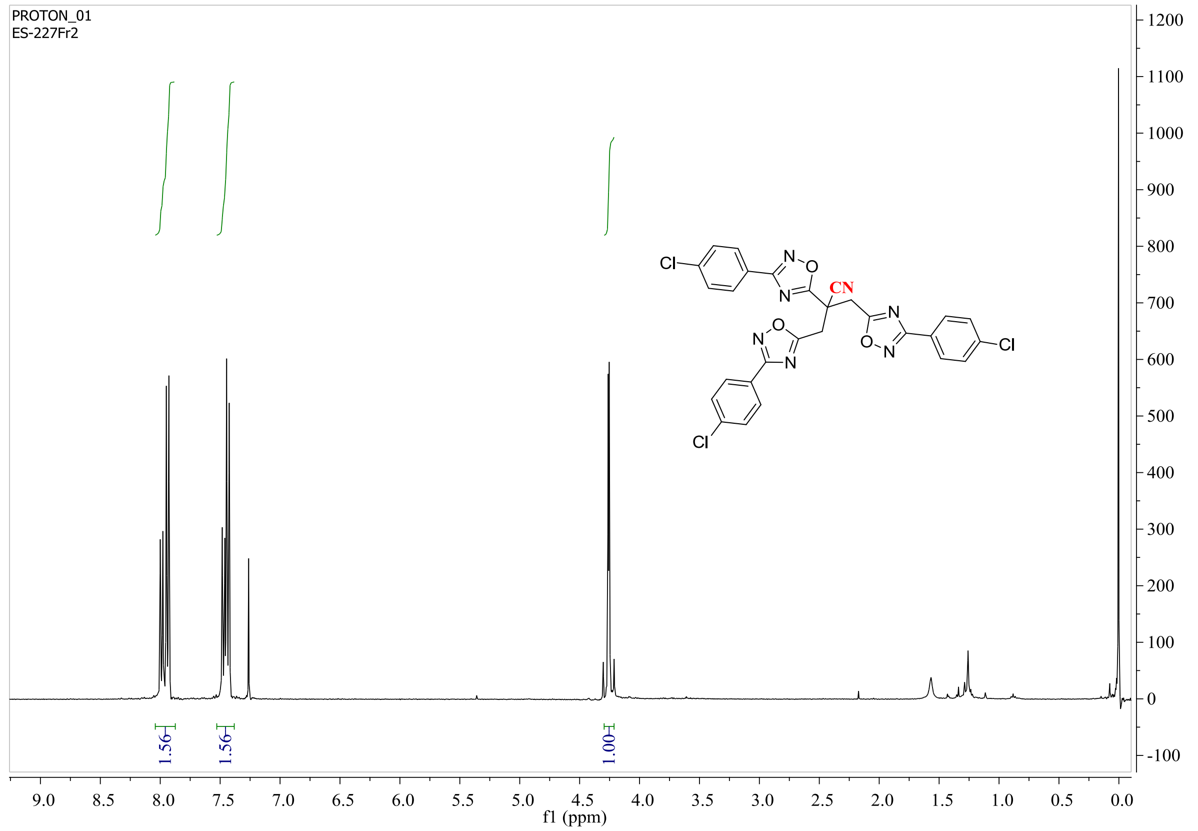

^{13}C NMR Spectrum of **3b**

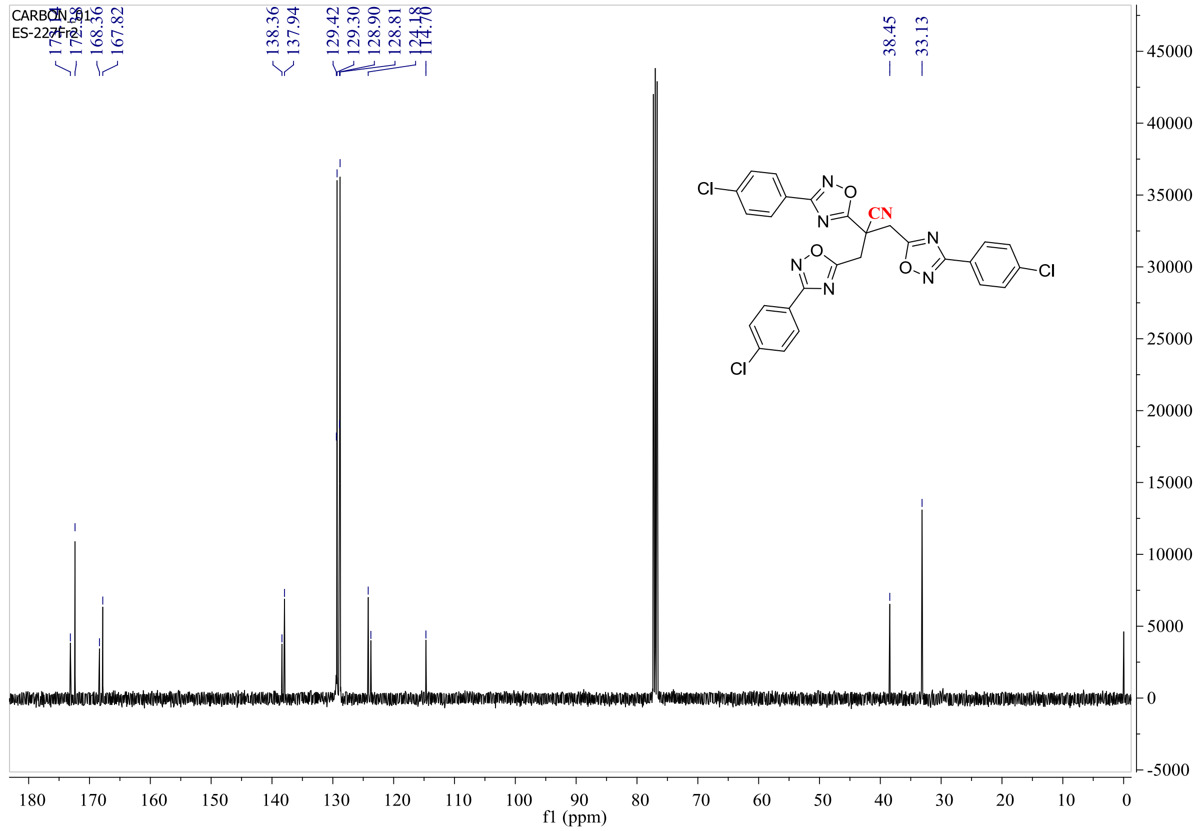

^{1}H NMR Spectrum of **3c**

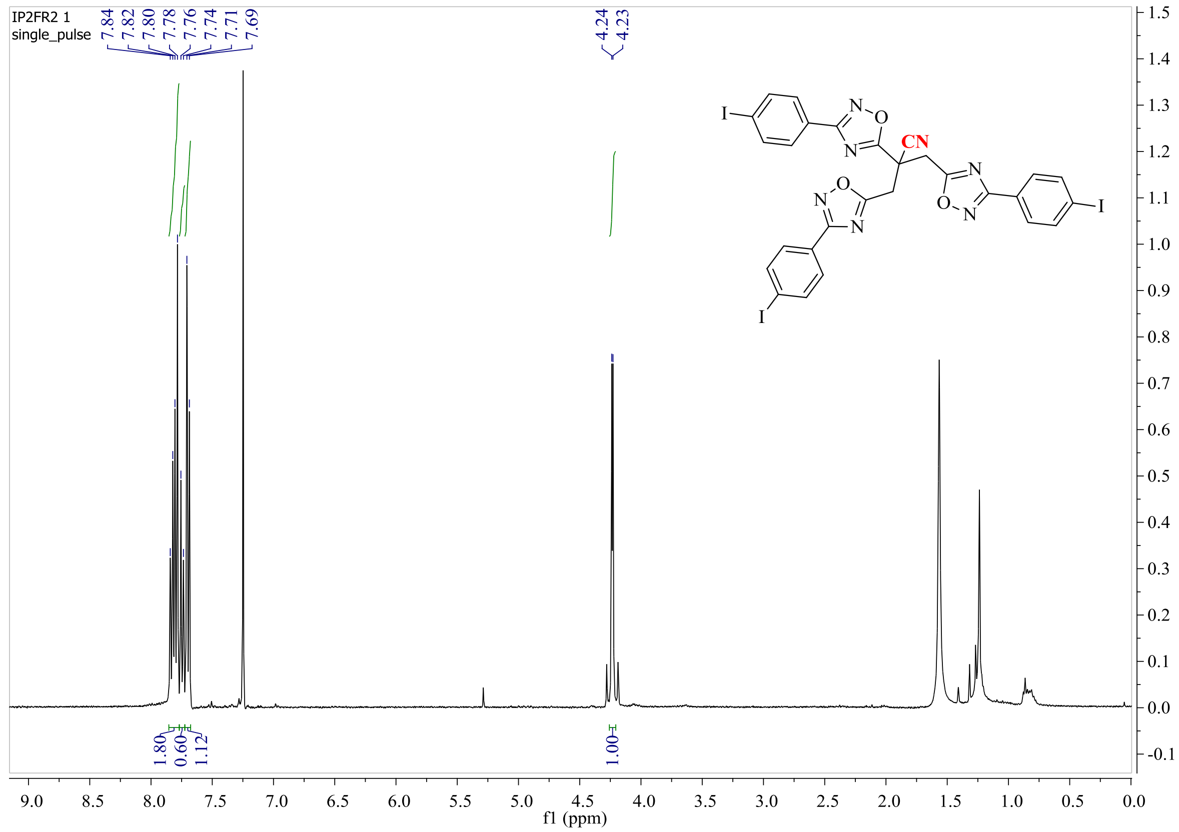

^{13}C NMR Spectrum of **3c**

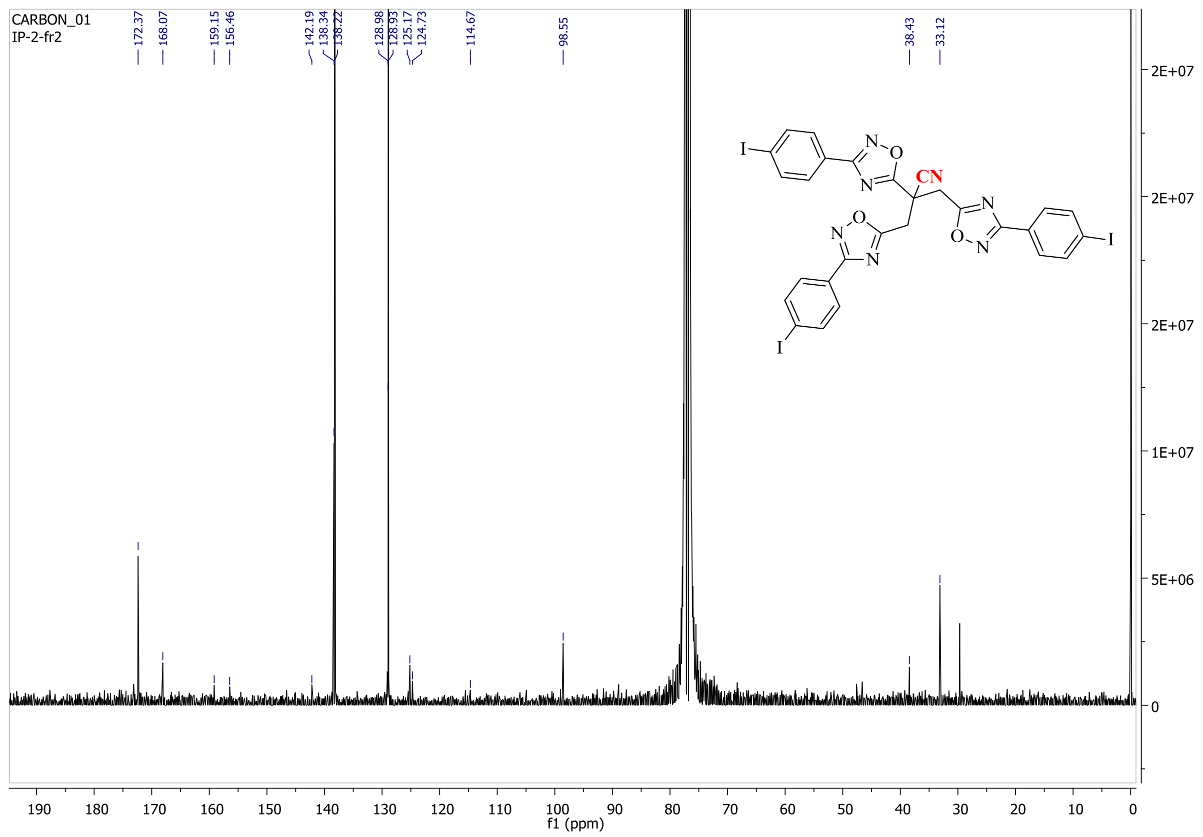

^{1}H NMR Spectrum of **3d**

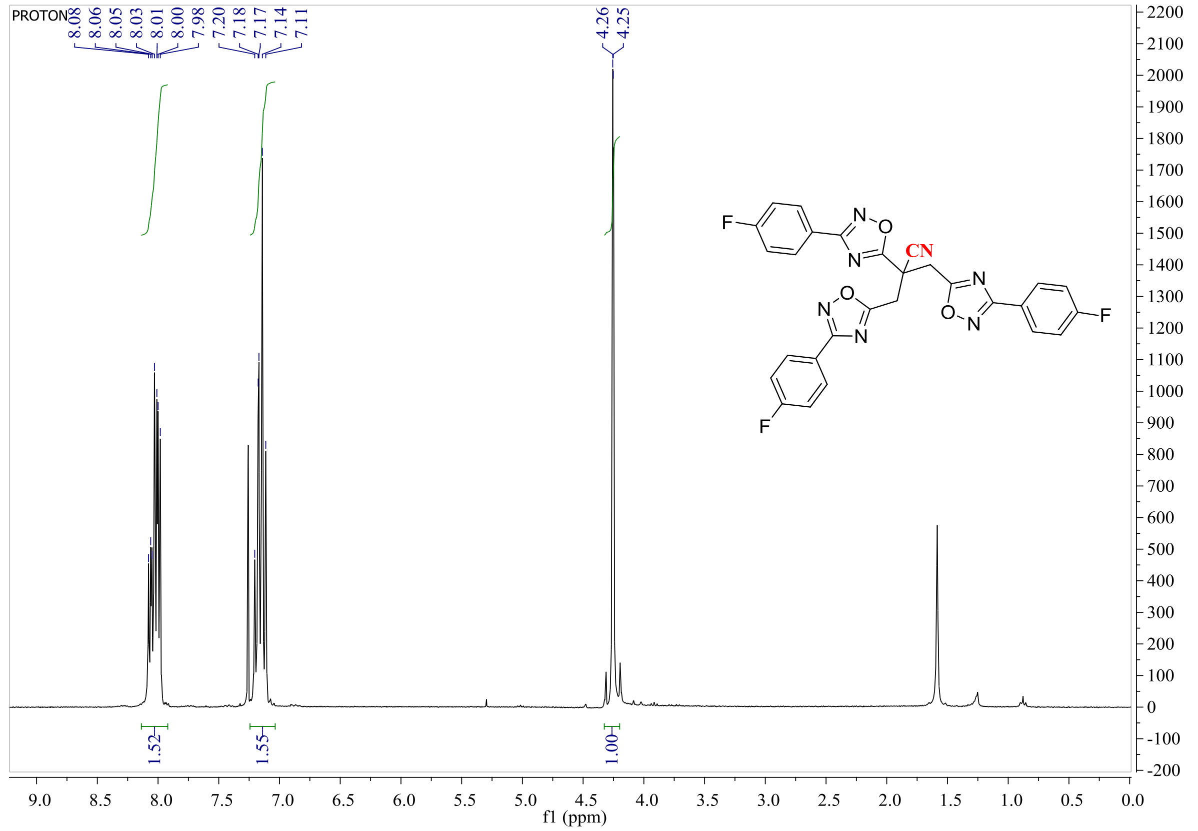

^{13}C NMR Spectrum of **3d**

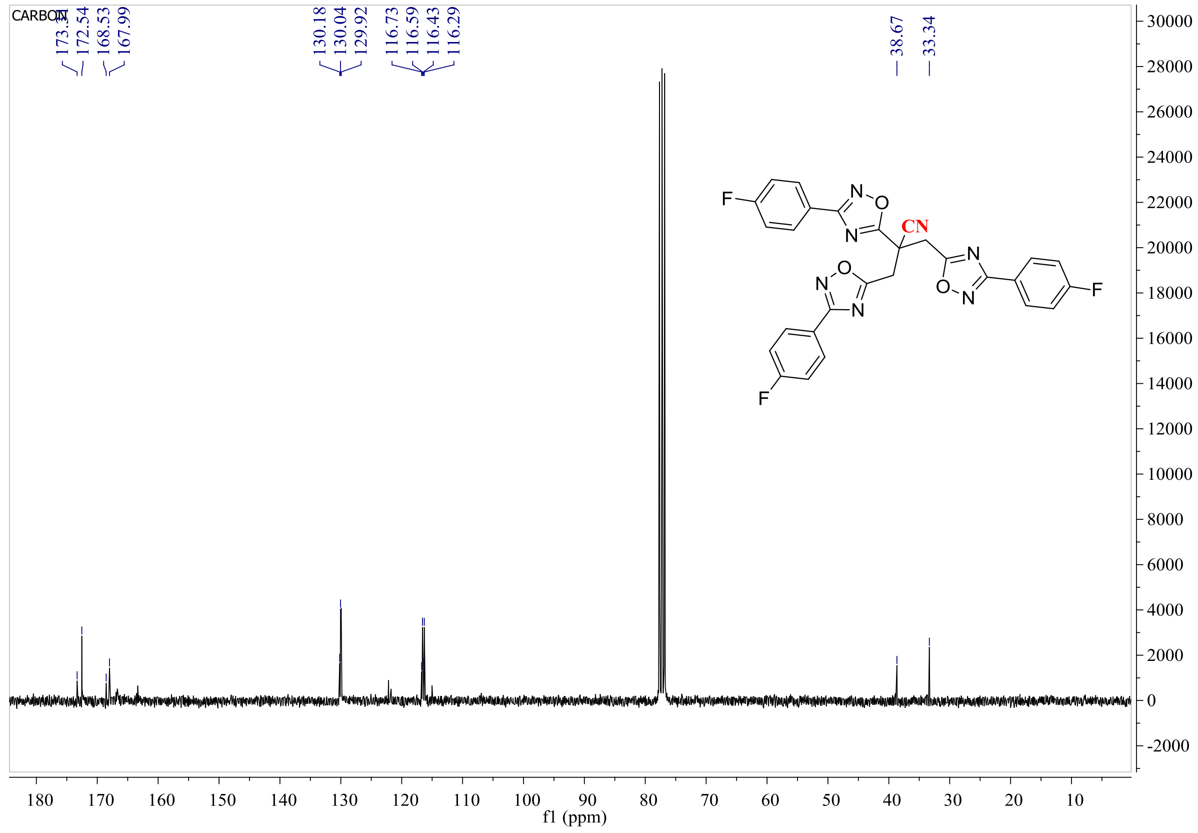

^{1}H NMR Spectrum of **3e**

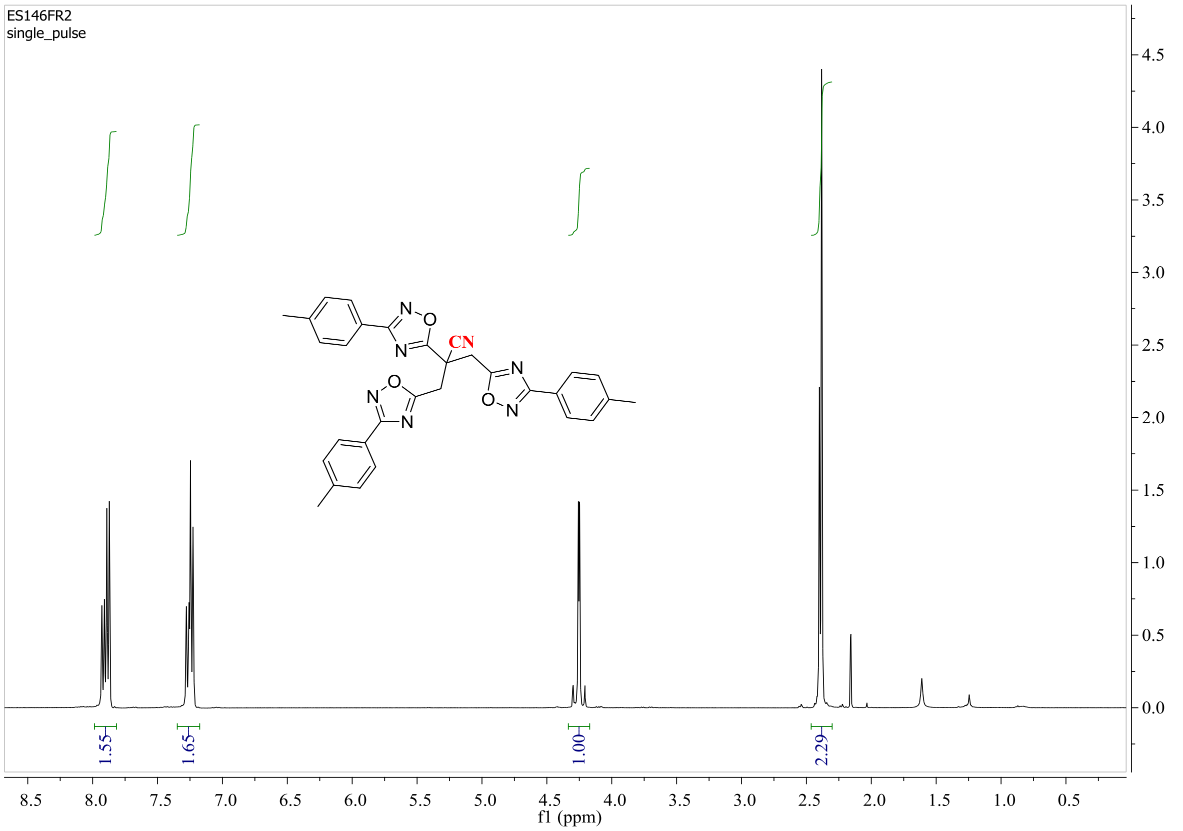

^{13}C NMR Spectrum of **3e**

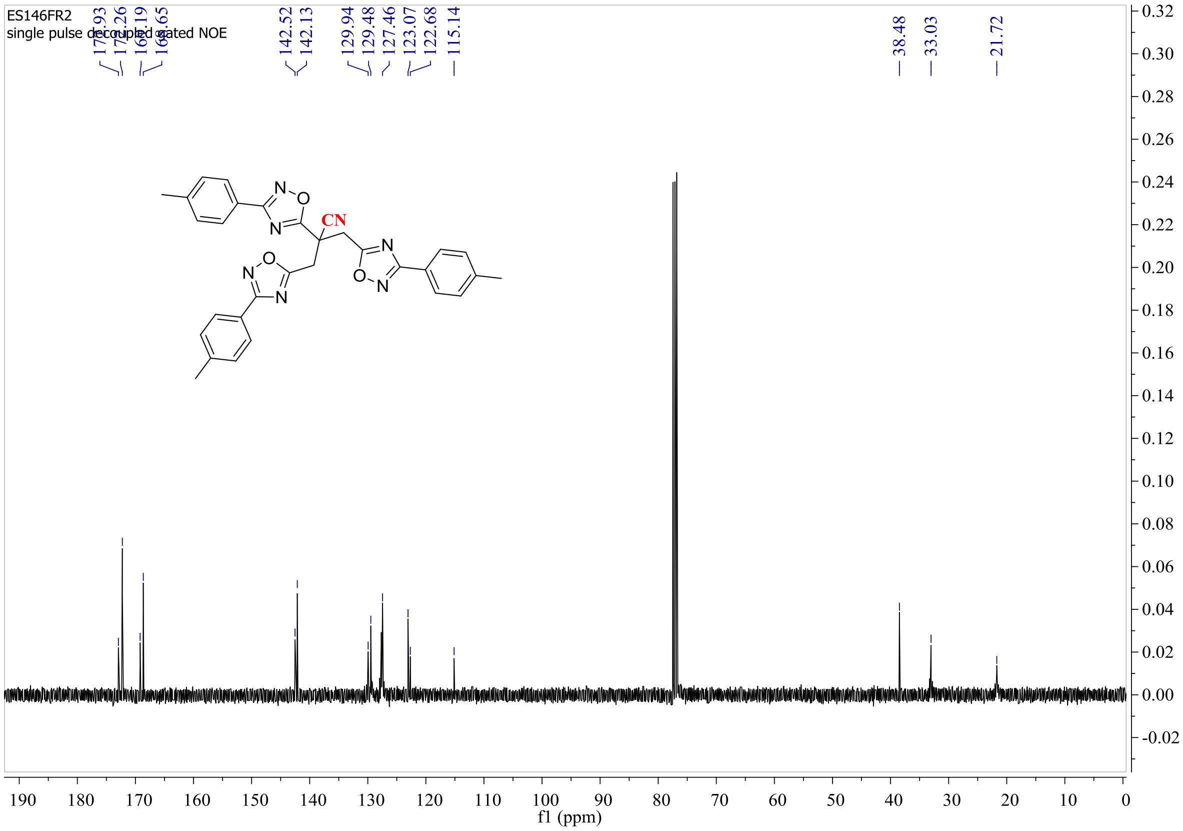

^{1}H NMR Spectrum of **3f**

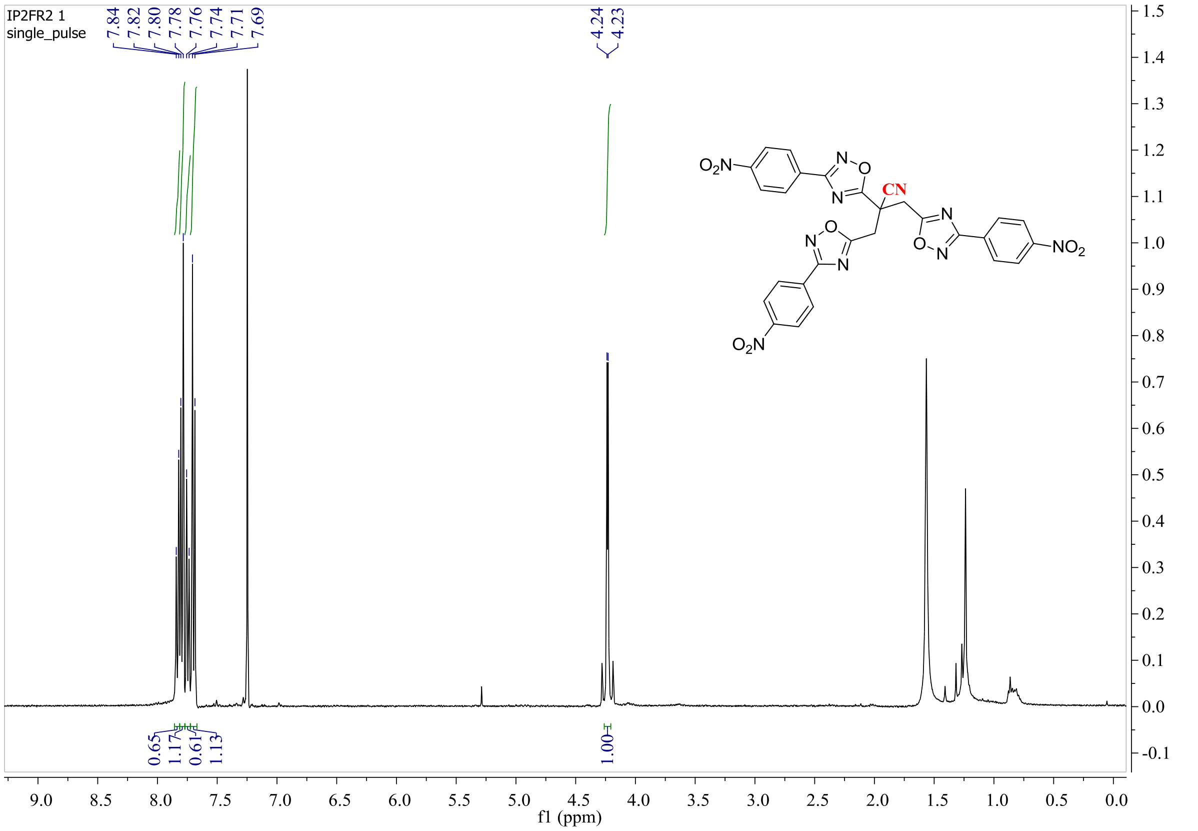

^{13}C NMR Spectrum of **3f**

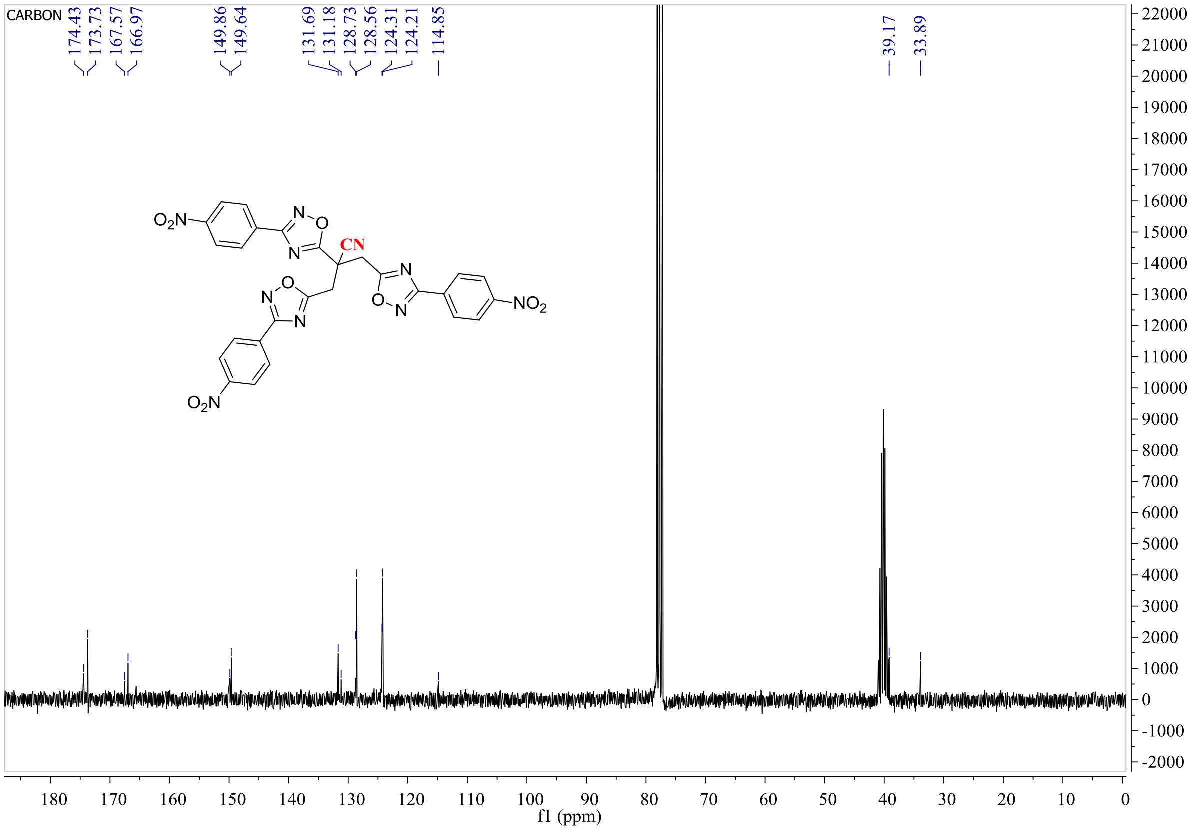

^{1}H NMR Spectrum of **3g**

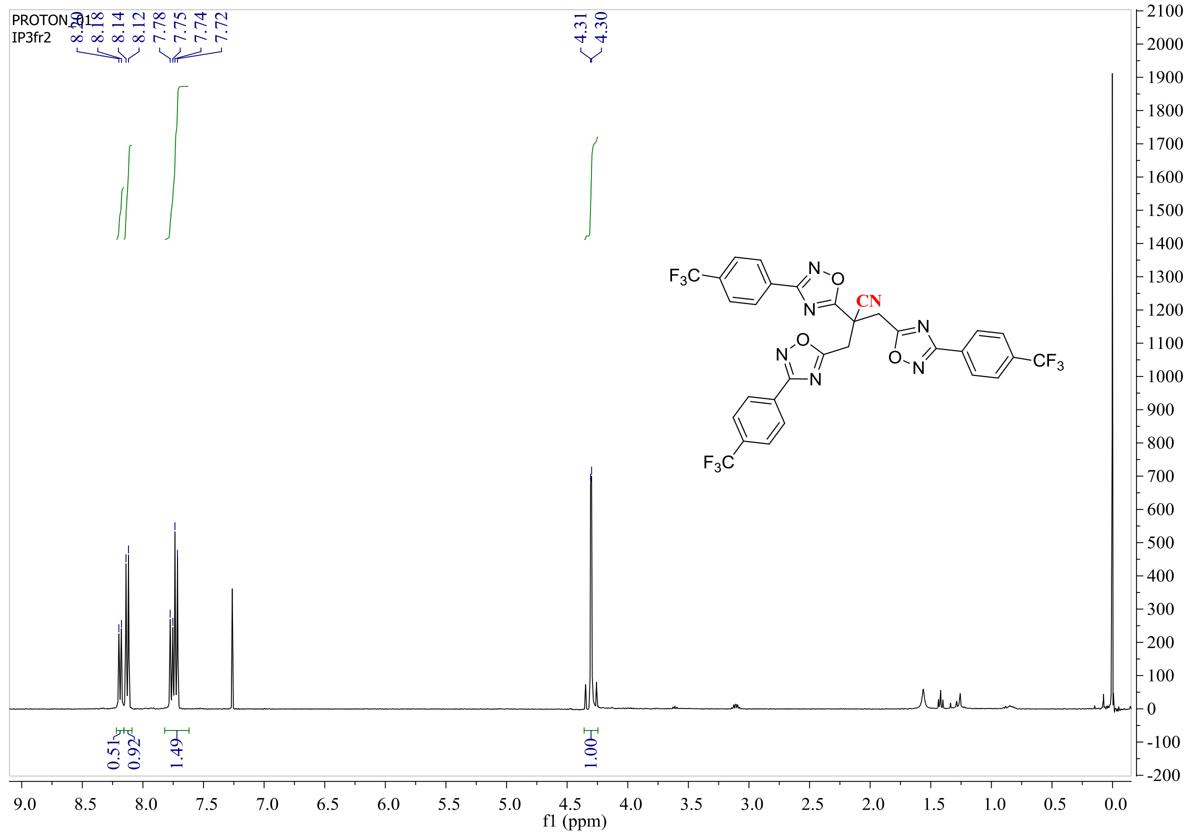

^{13}C NMR Spectrum of **3g**

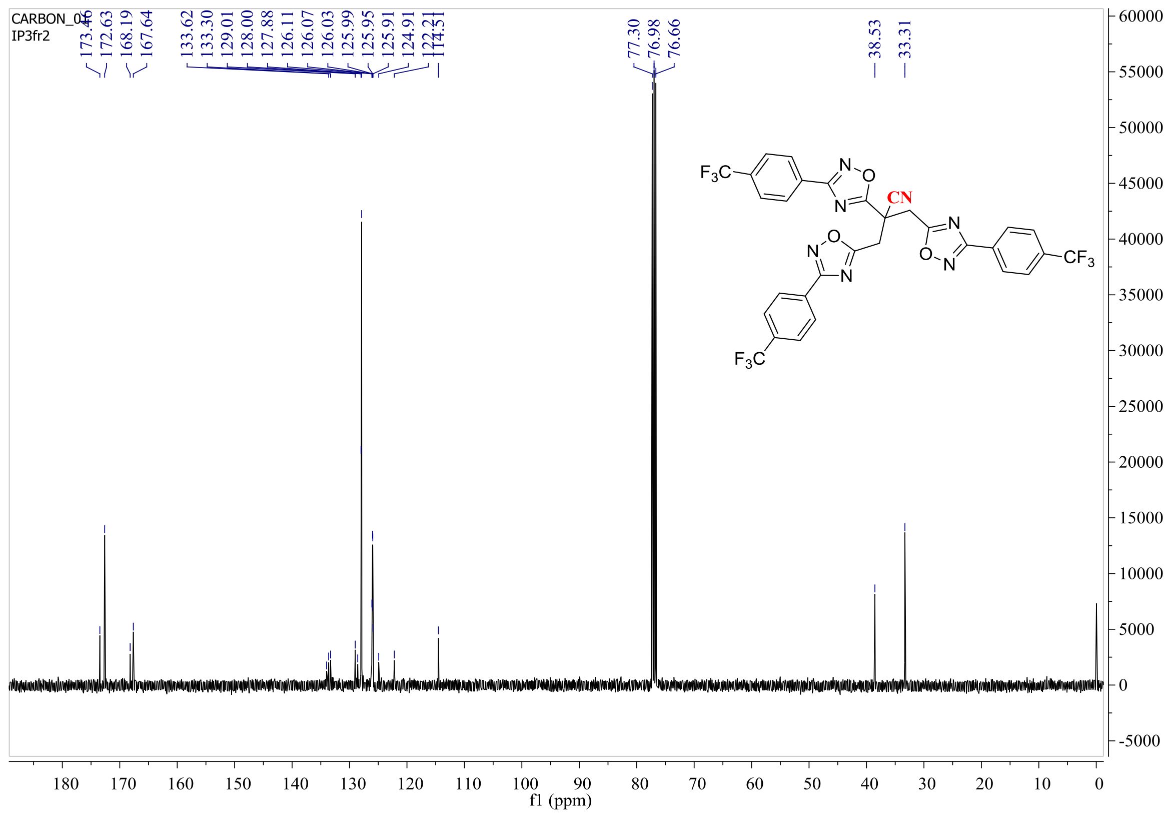

^{1}H NMR Spectrum of **3h**

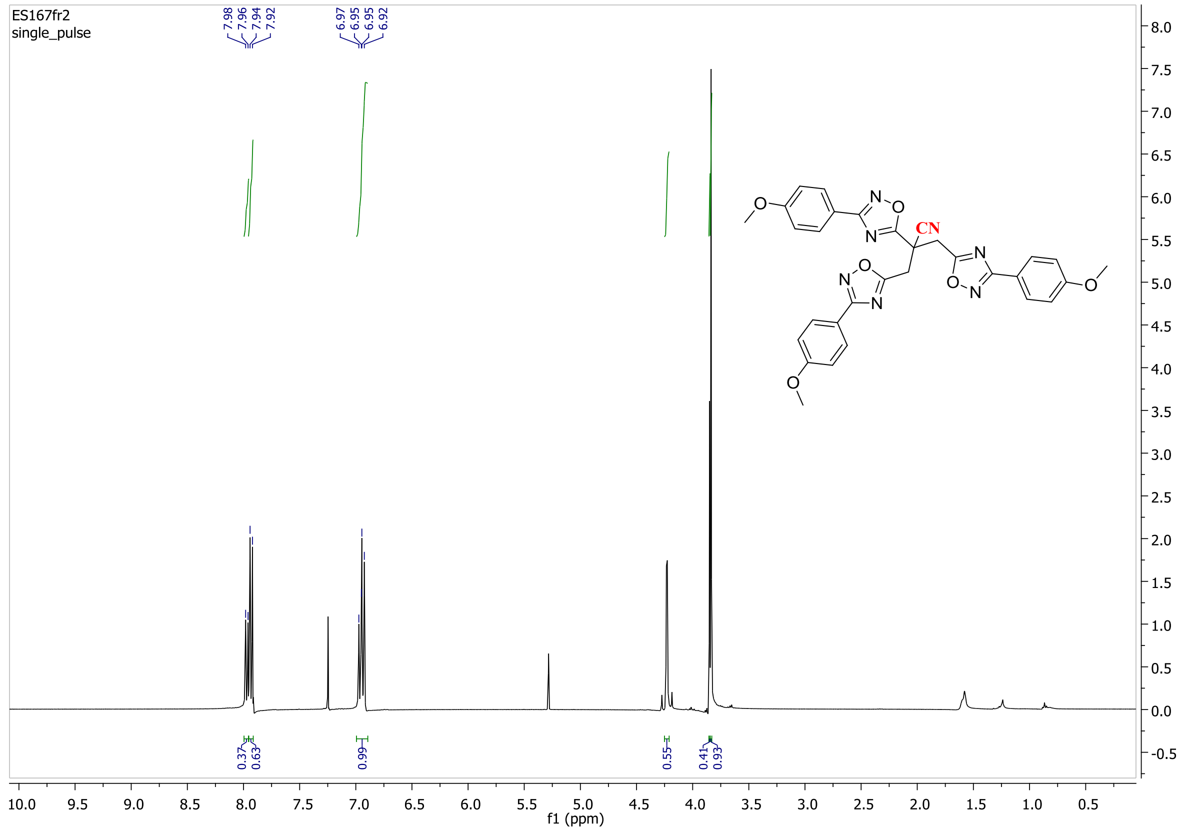

^{13}C NMR Spectrum of **3h**

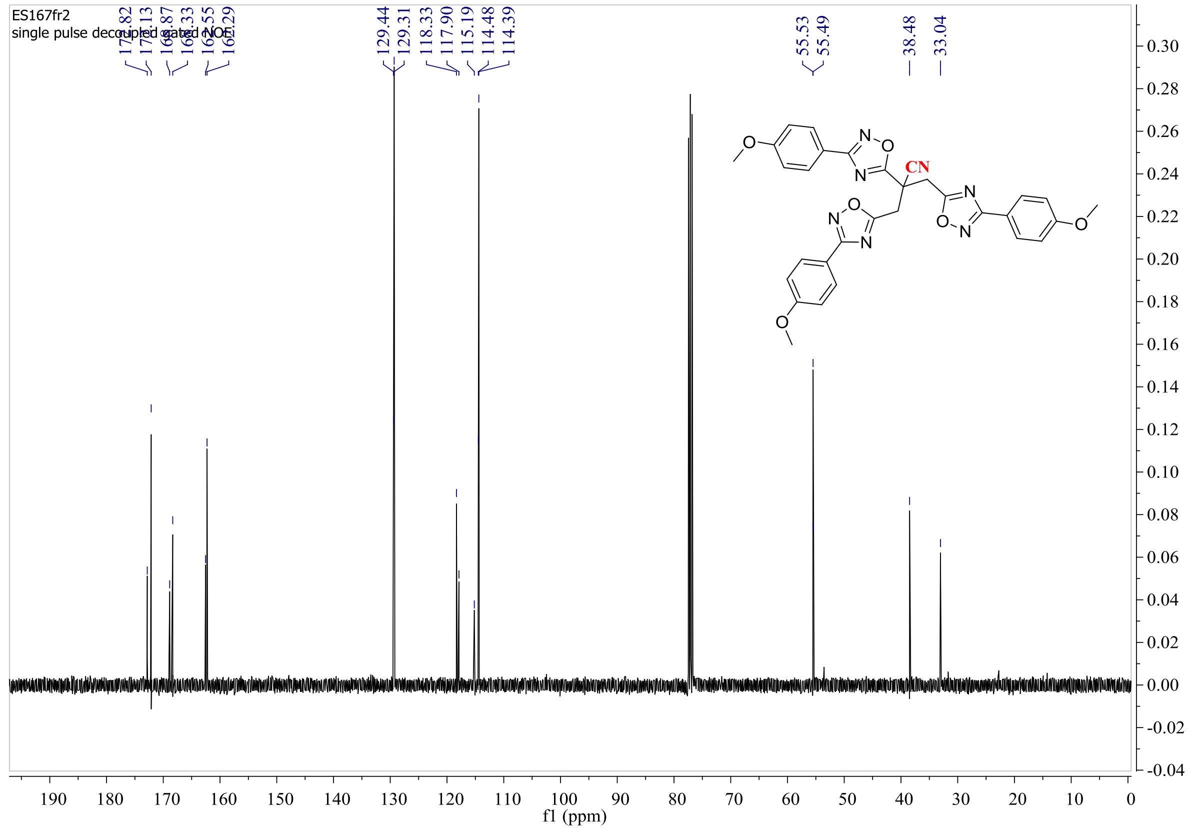

^{1}H NMR Spectrum of **3j**

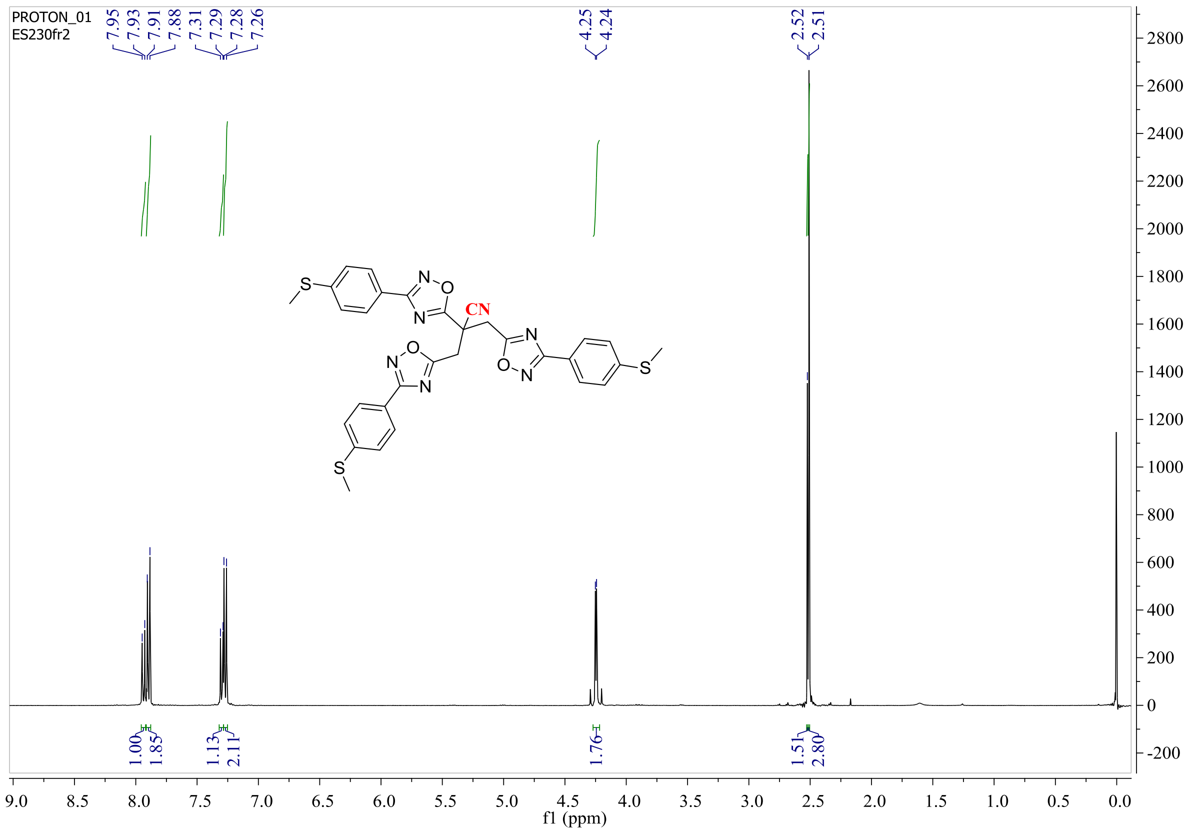

^{13}C NMR Spectrum of **3j**

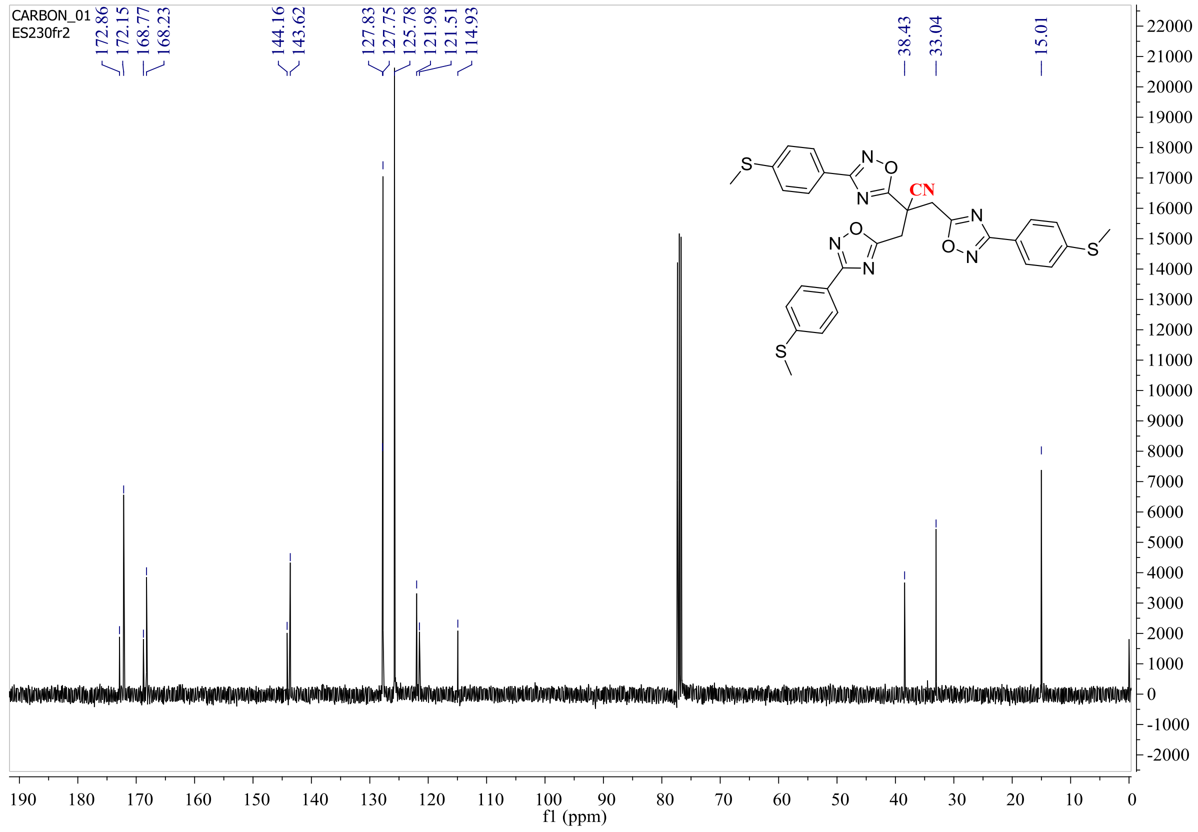

^{1}H and ^{13}C NMR SPECTRA of 4a-j

^{1}H NMR Spectrum of **4a**

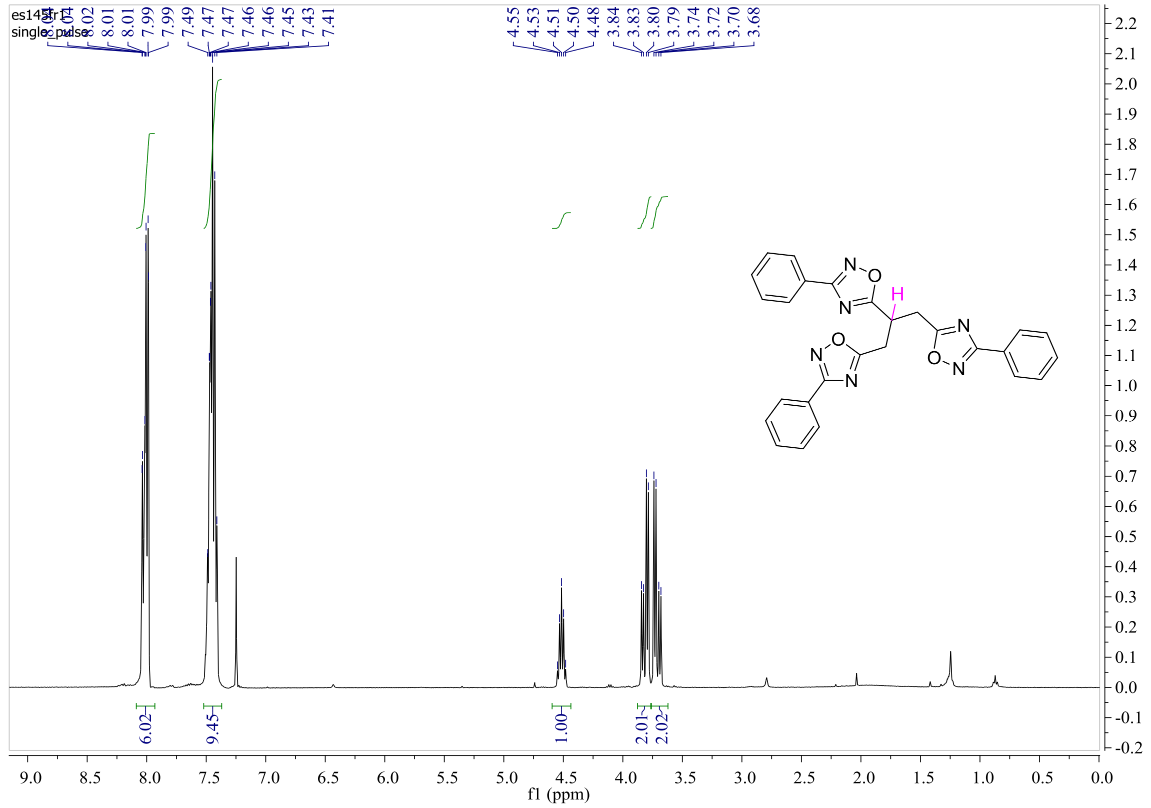

^{13}C NMR Spectrum of **4a**

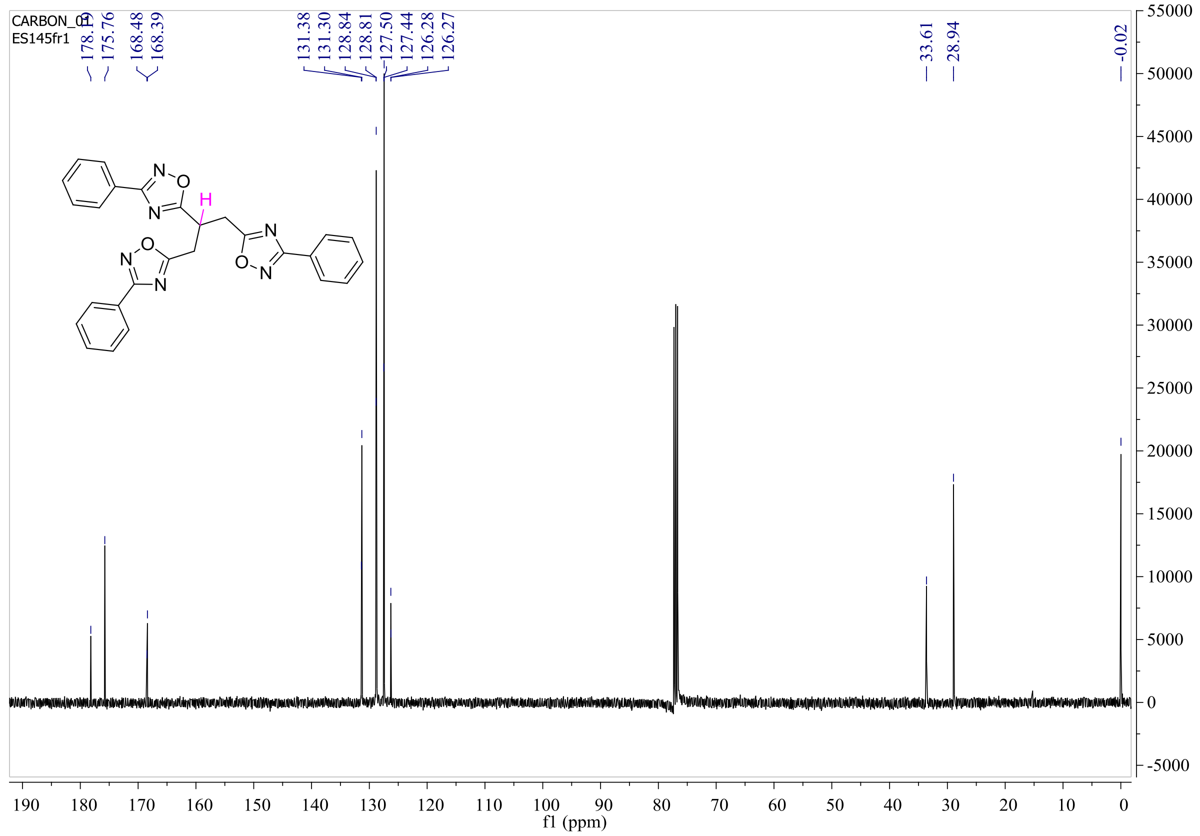

^{1}H NMR Spectrum of **4b**

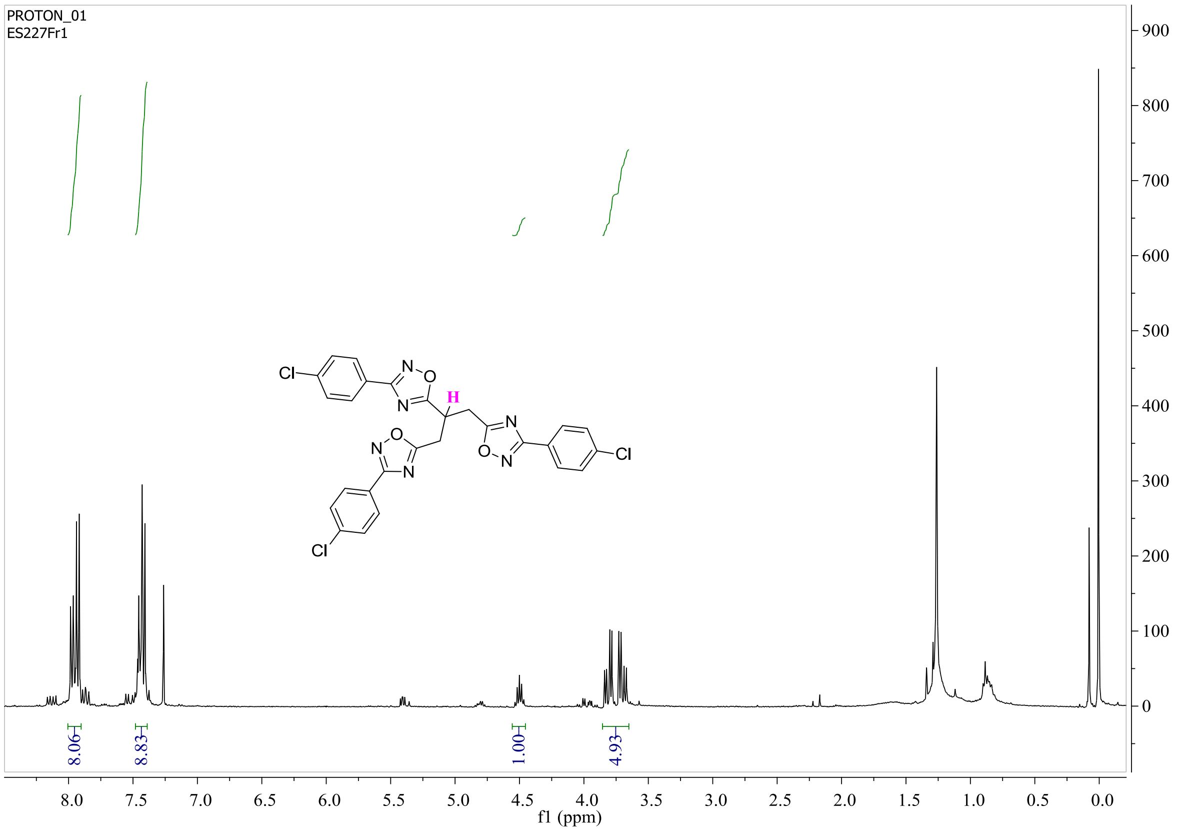

^{13}C NMR Spectrum of **4b**

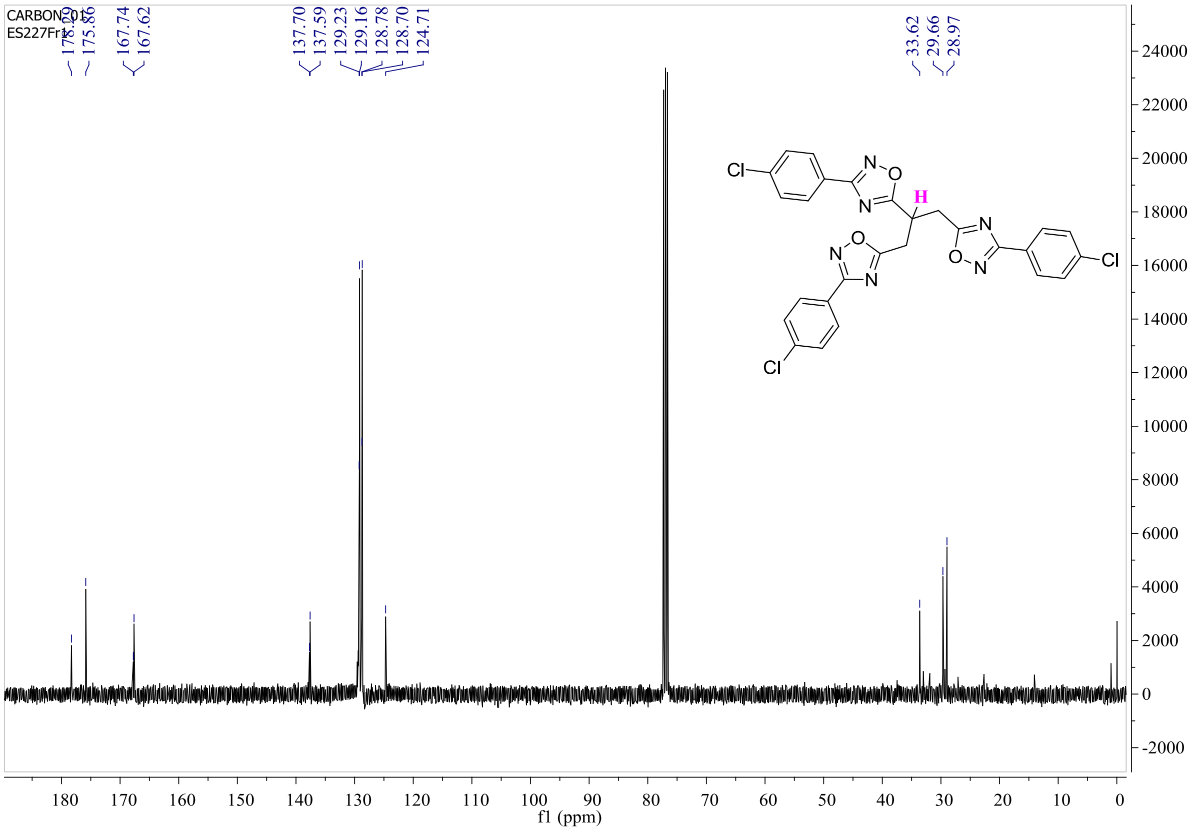

^{1}H NMR Spectrum of **4c**

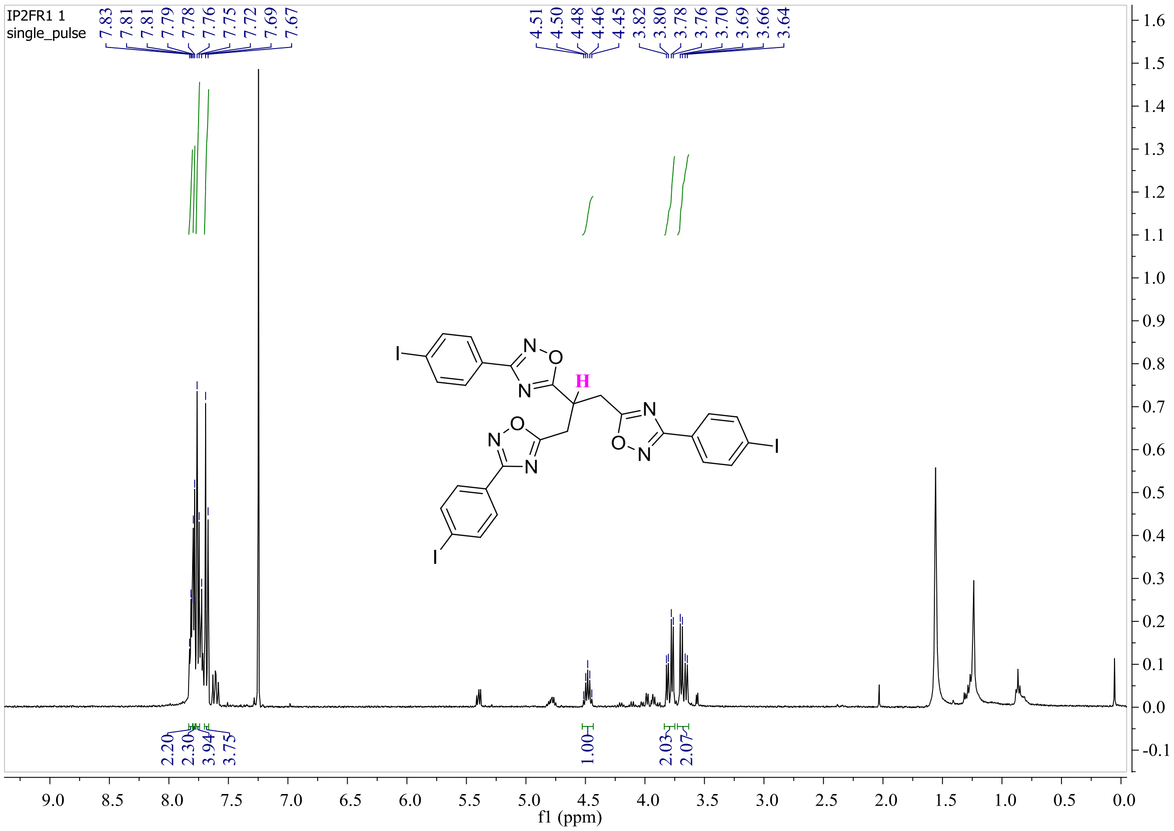

^{13}C NMR Spectrum of **4c**

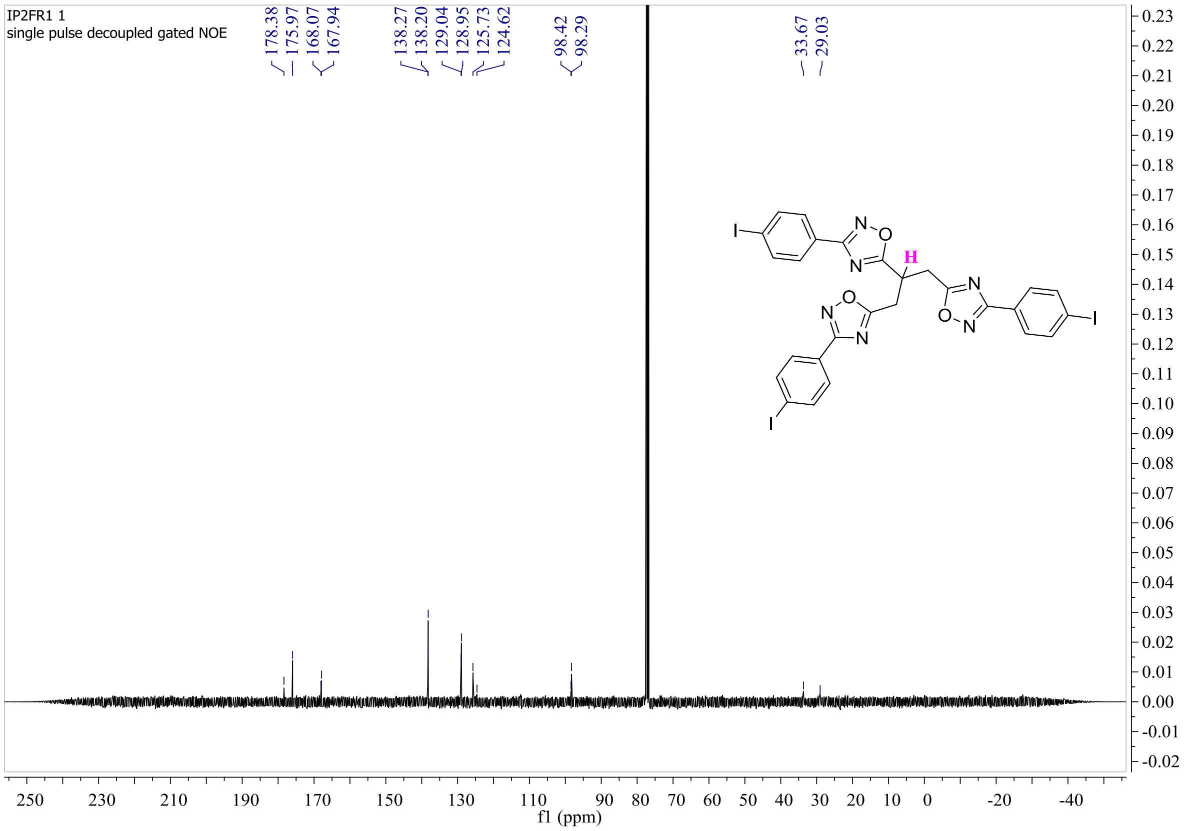

^{1}H NMR Spectrum of **4d**

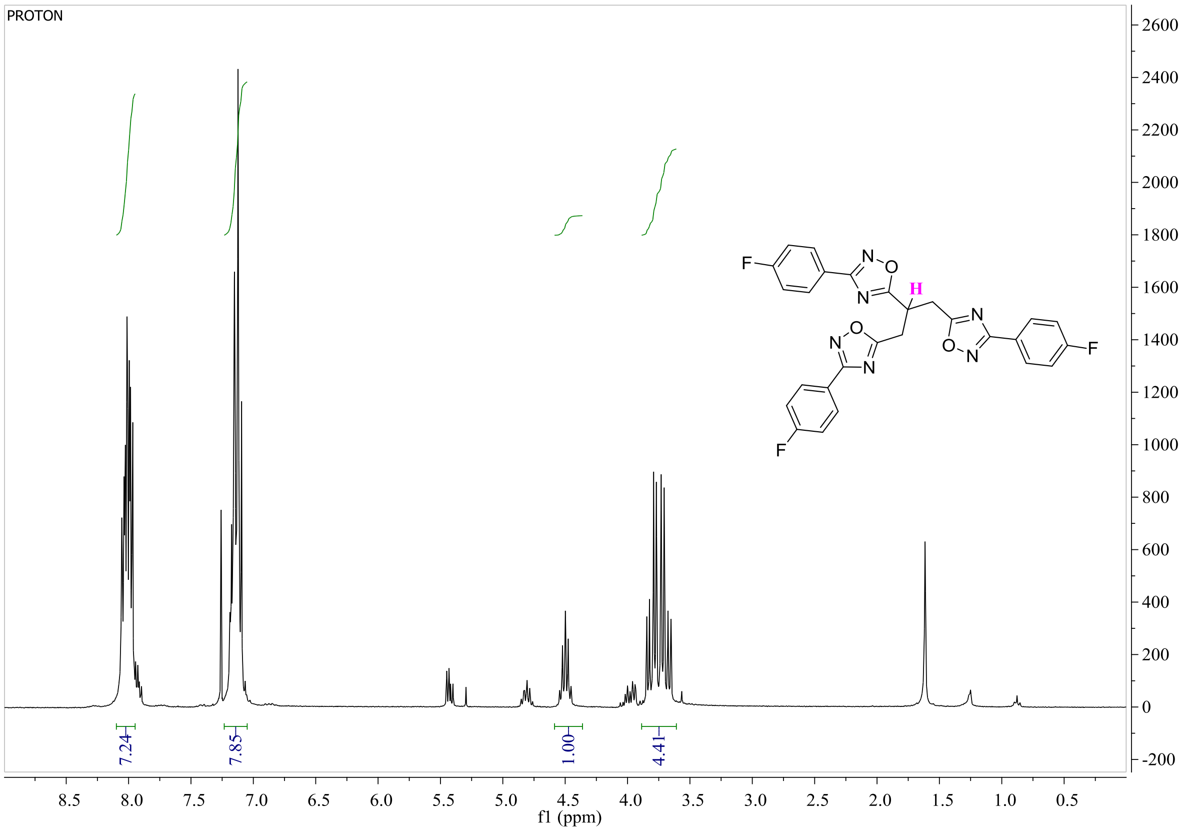

^{13}C NMR Spectrum of **4d**

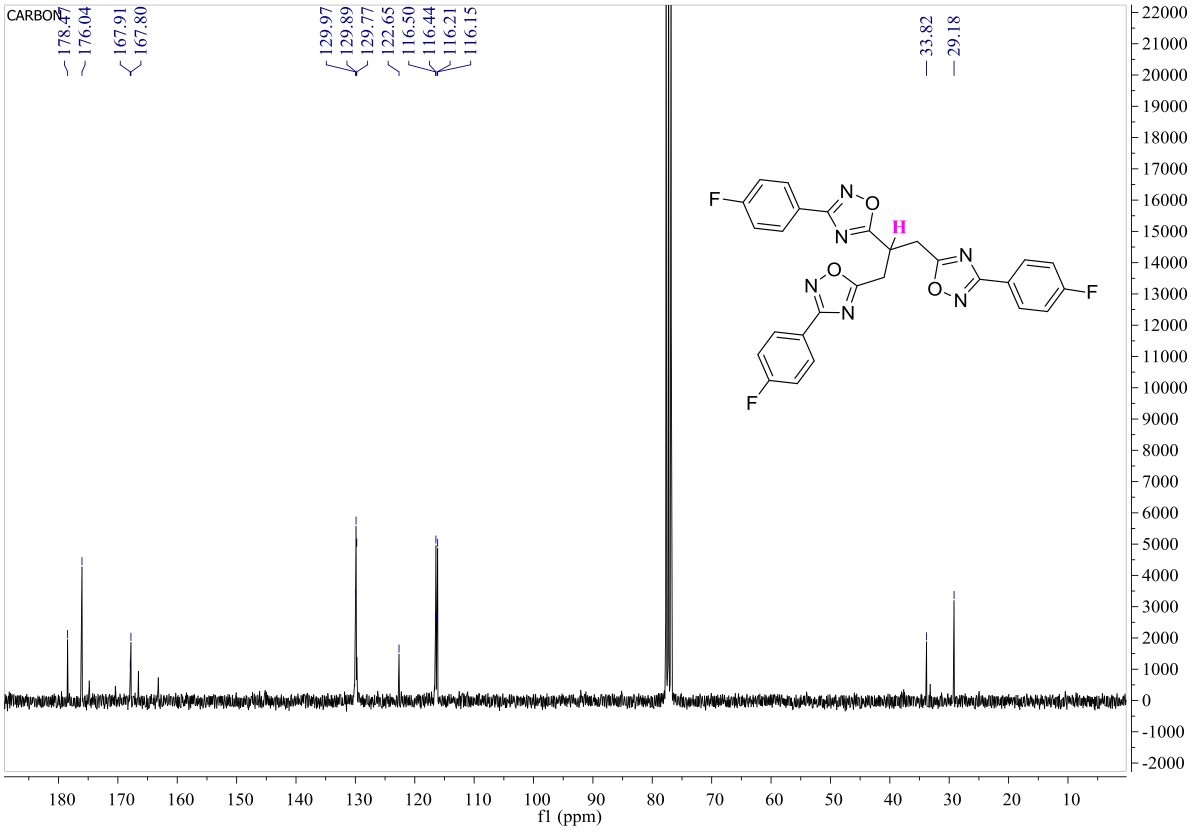

^{1}H NMR Spectrum of **4e**

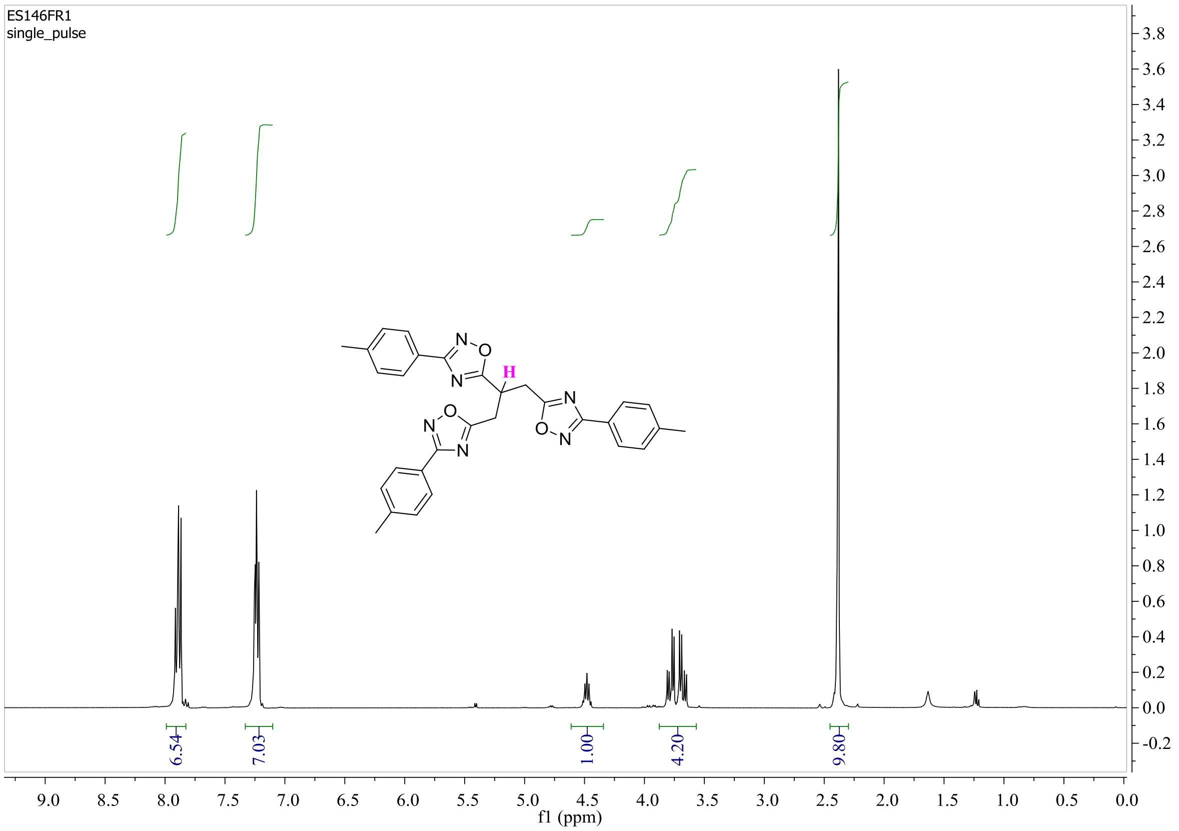

^{13}C NMR Spectrum of **4e**

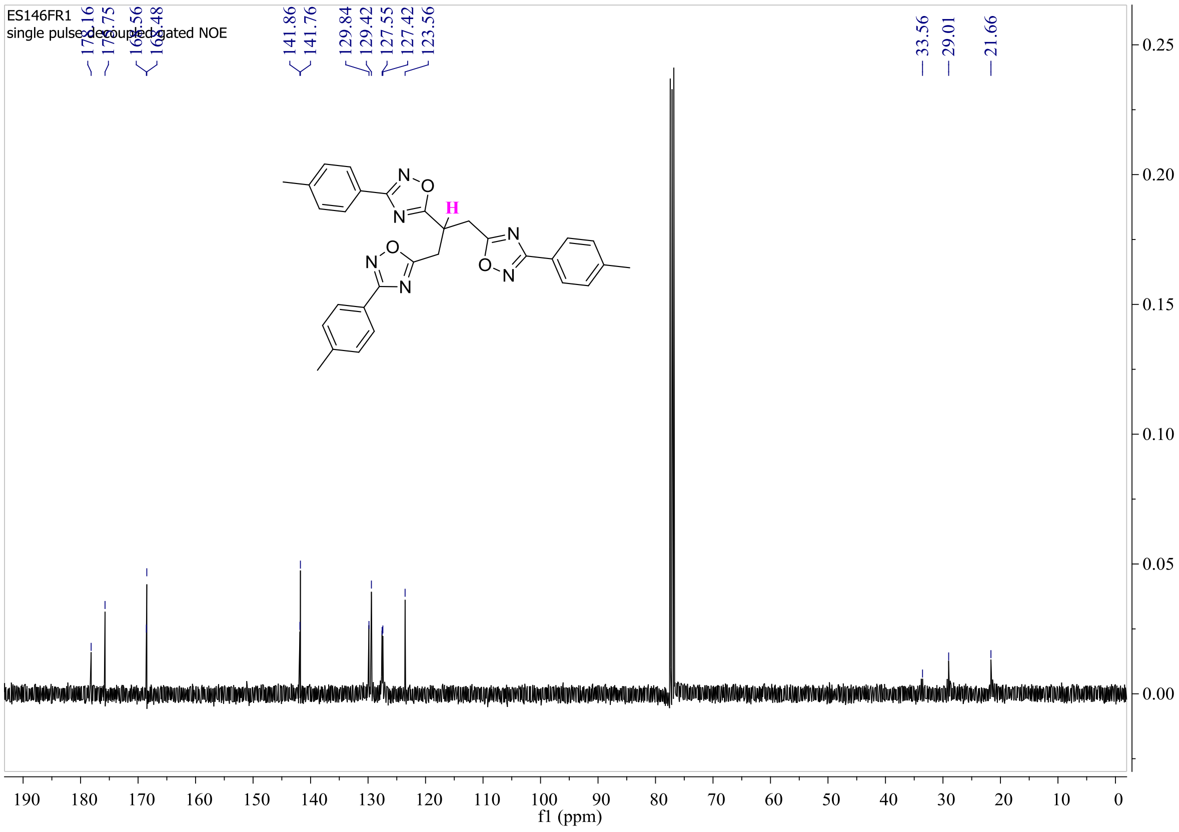

^{1}H NMR Spectrum of **4f**

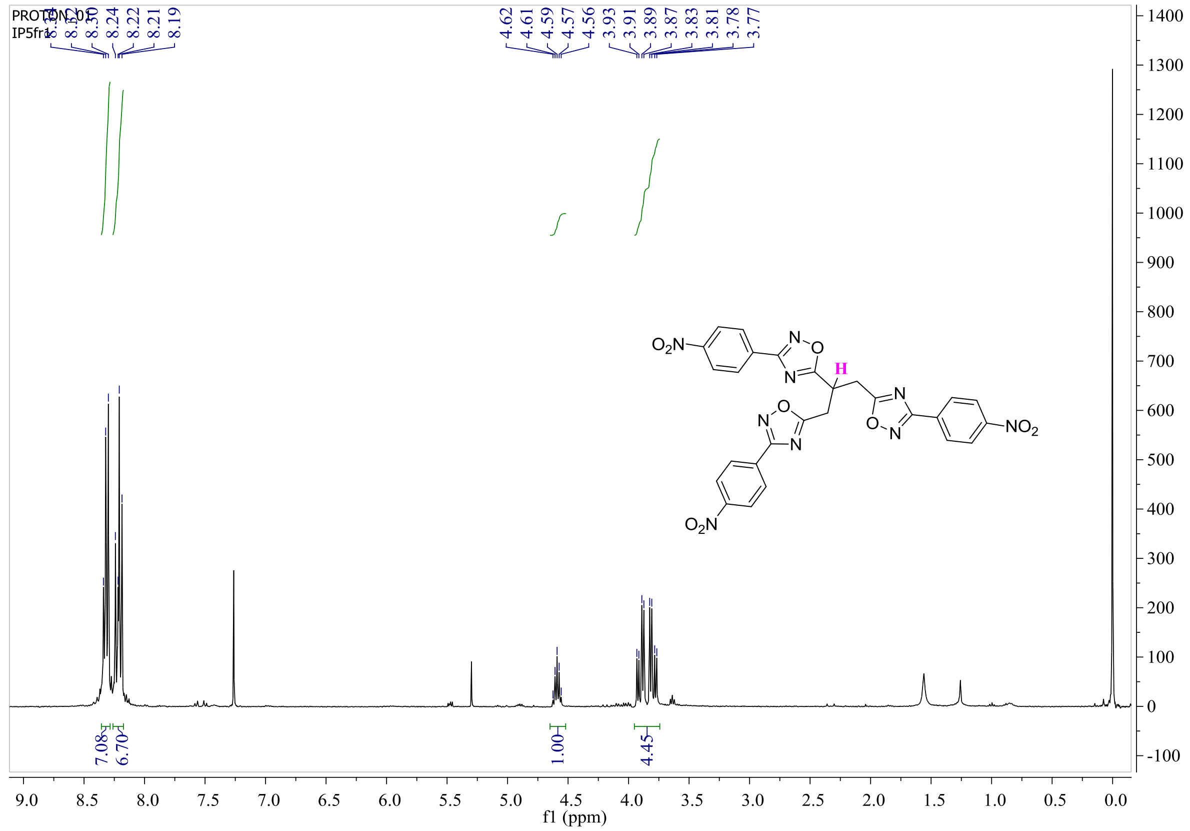

^{13}C NMR Spectrum of **4f**

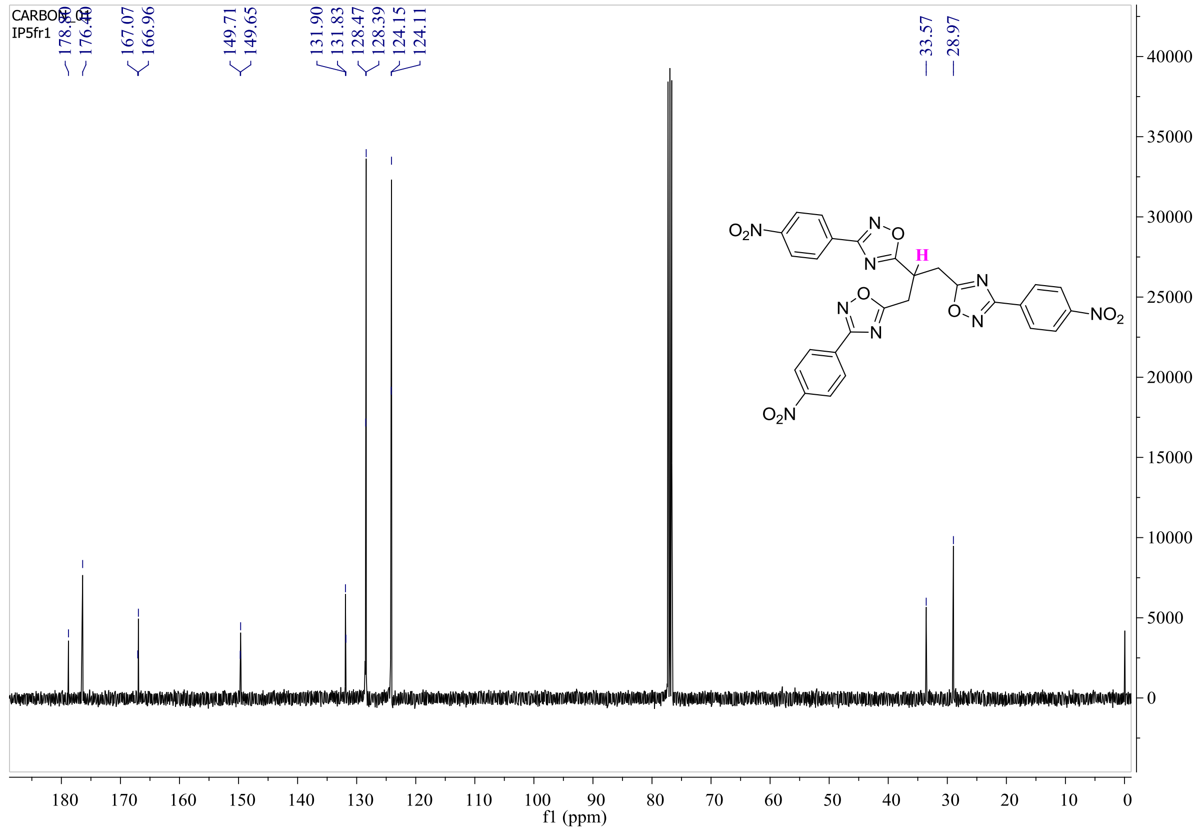

^{1}H NMR Spectrum of **4g**

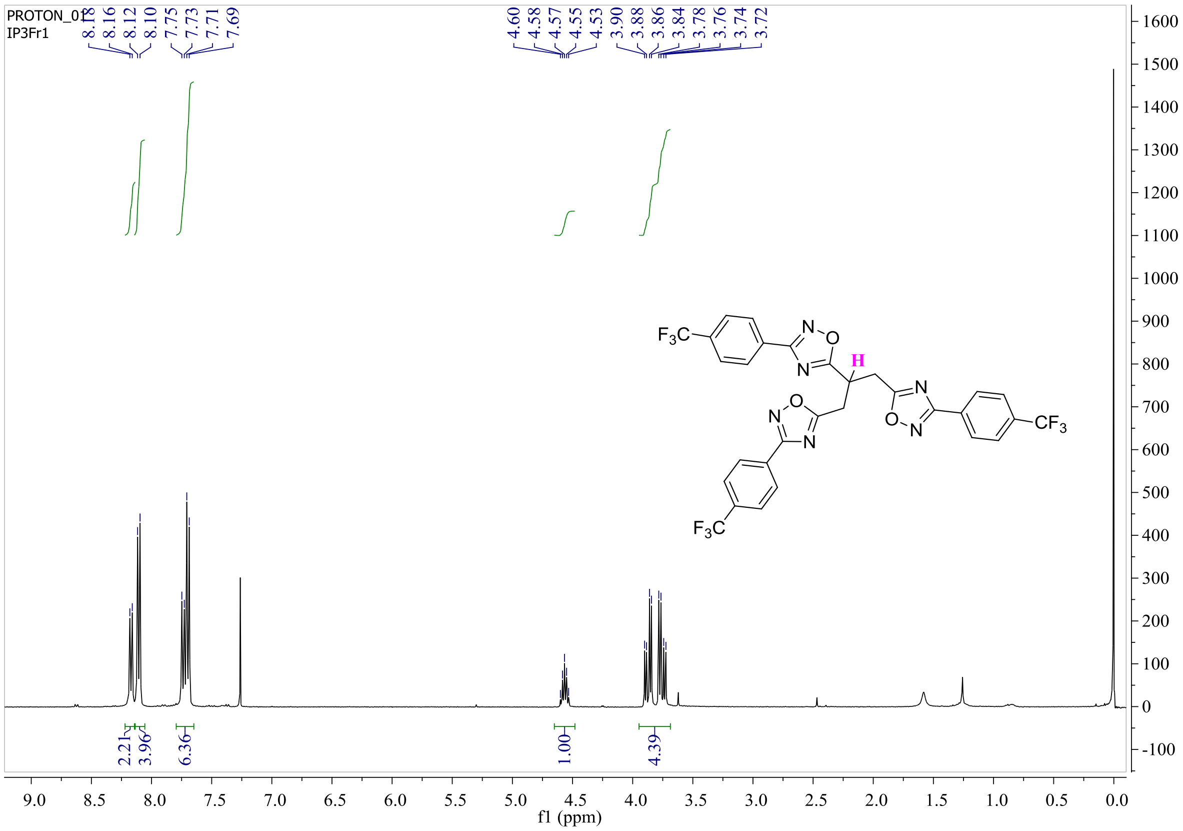

^{13}C NMR Spectrum of **4g**

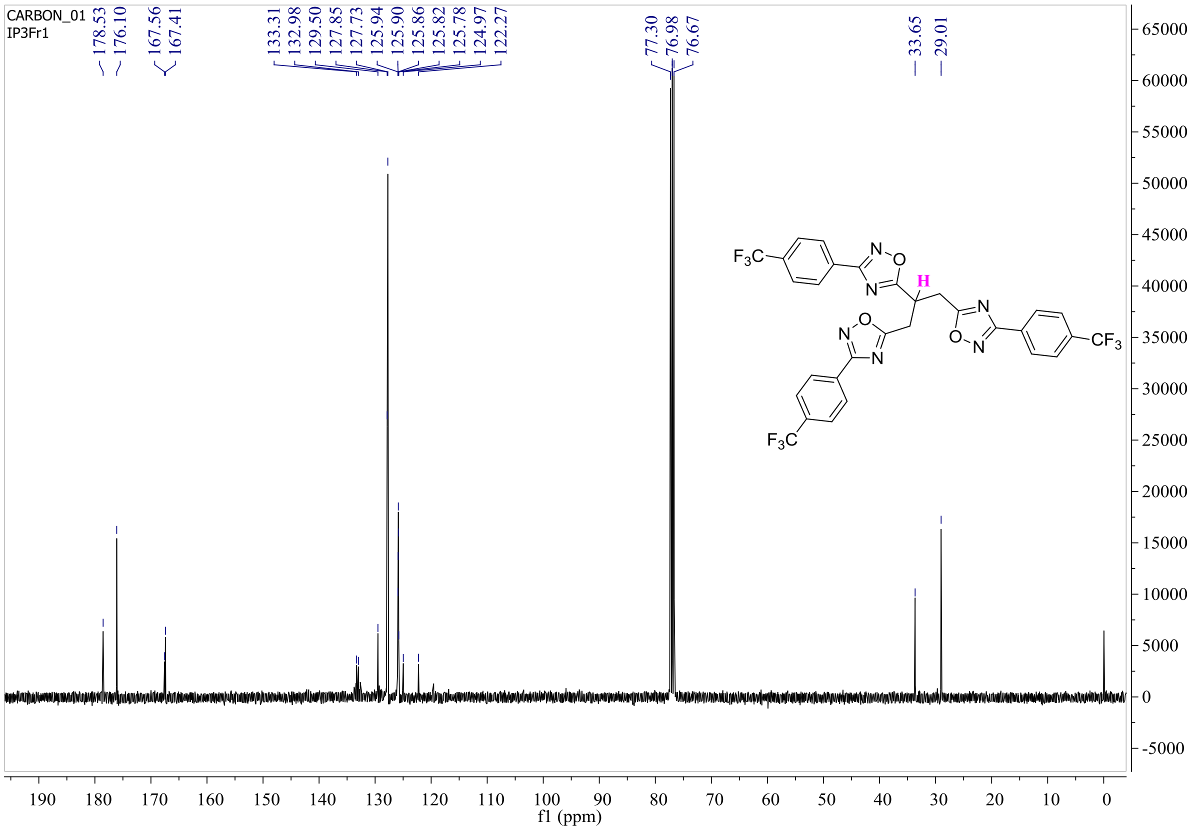

^{1}H NMR Spectrum of **4h**

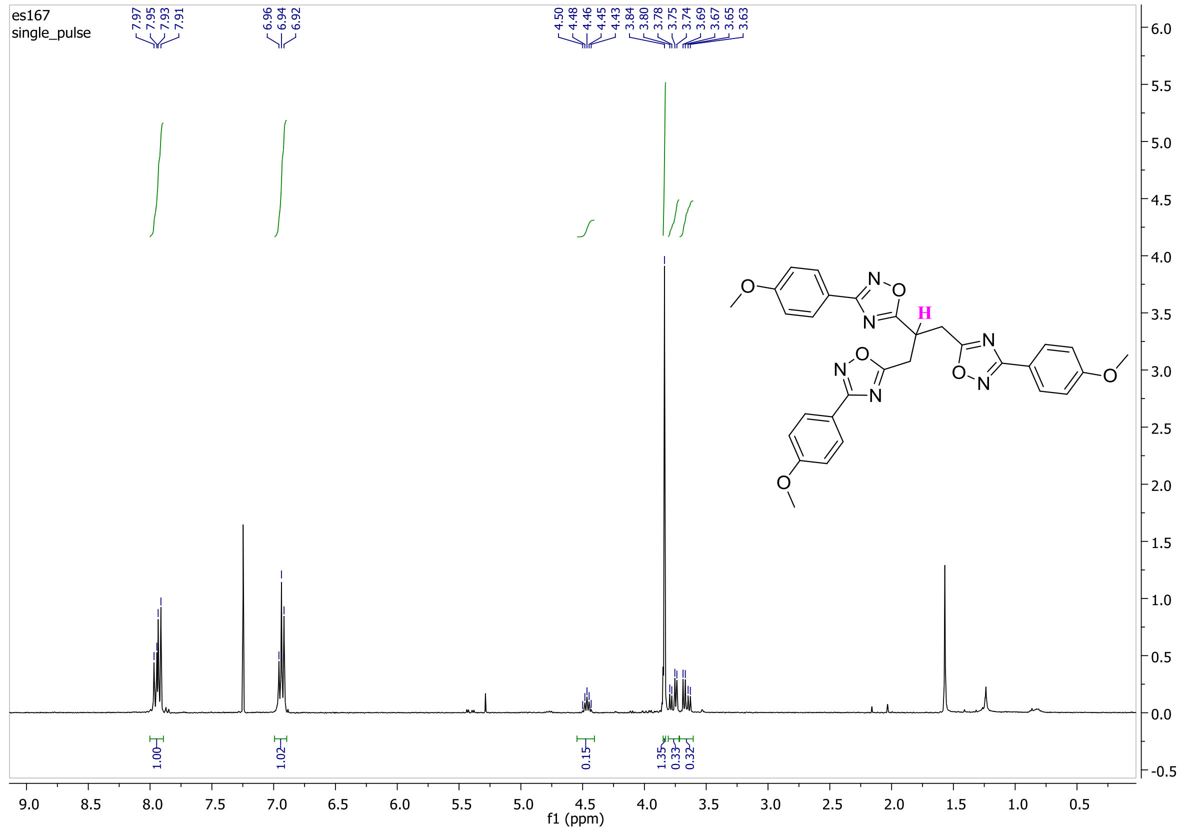

^{13}C NMR Spectrum of **4h**

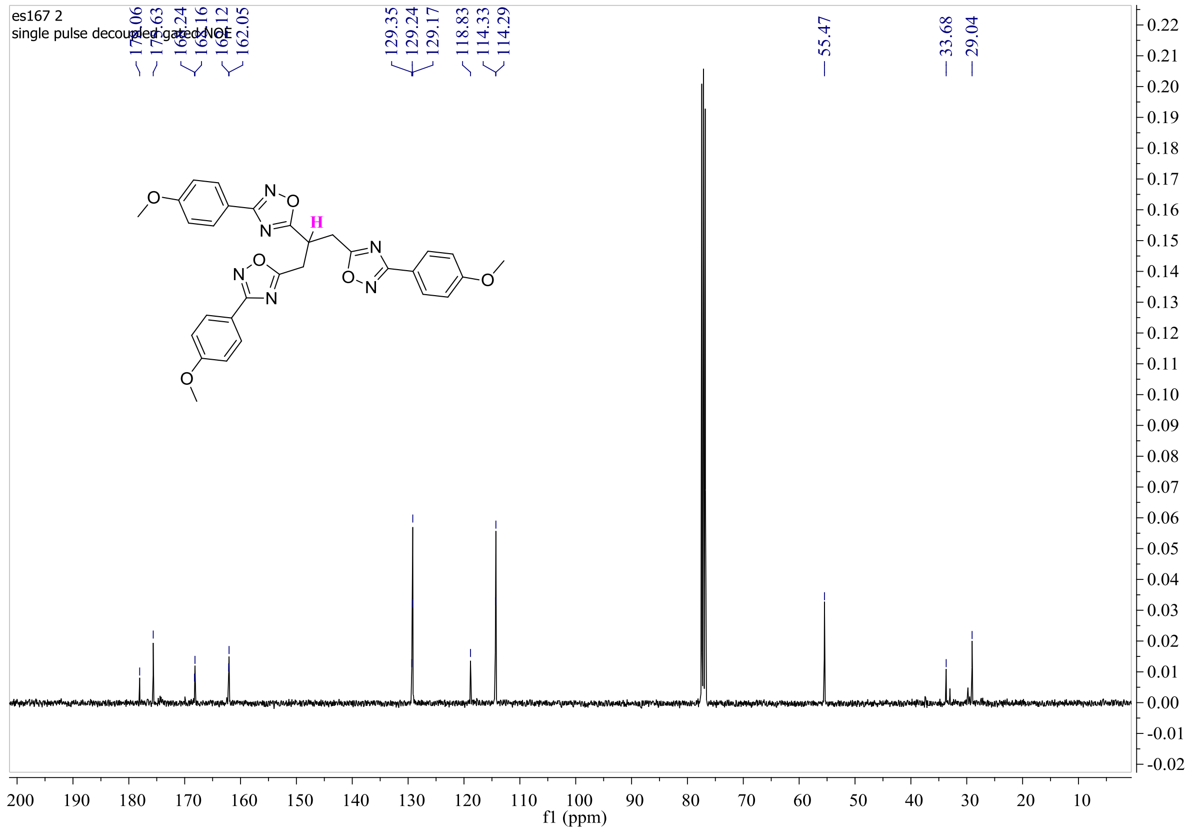

^{1}H NMR Spectrum of **4j**

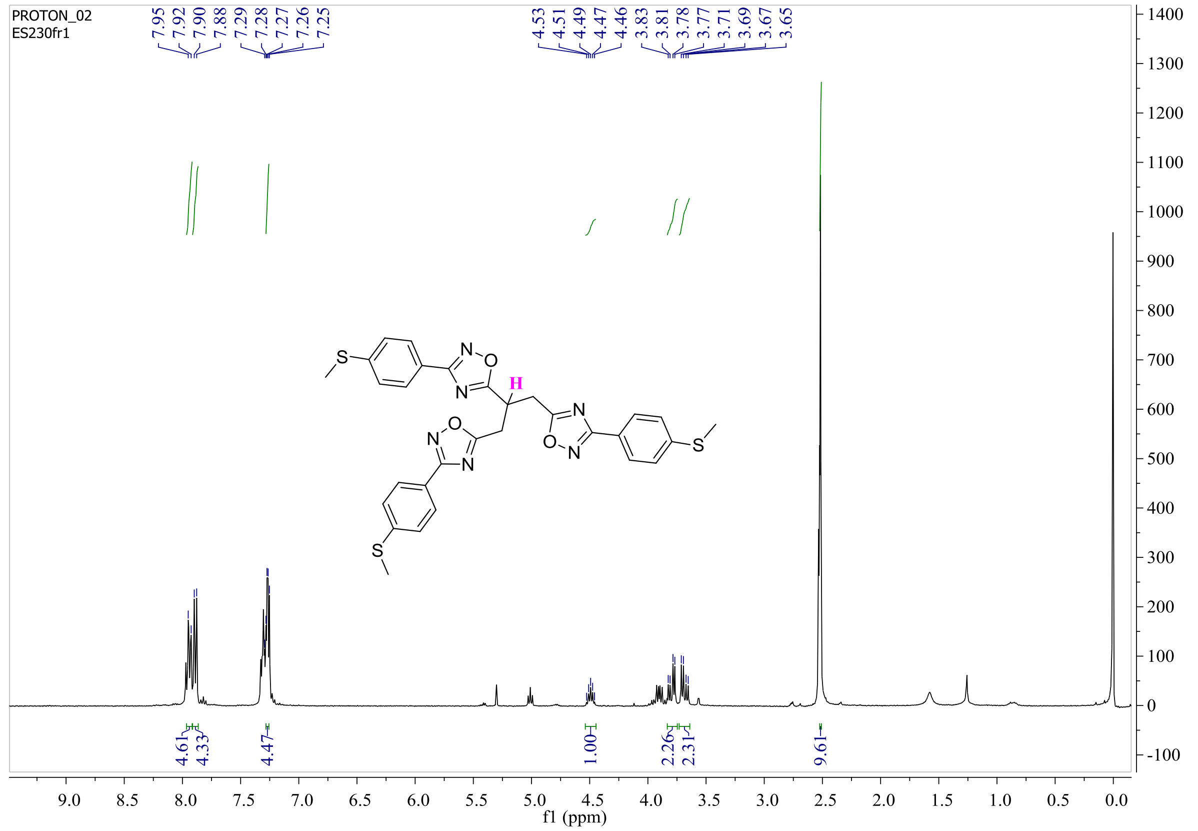

^{13}C NMR Spectrum of **4j**

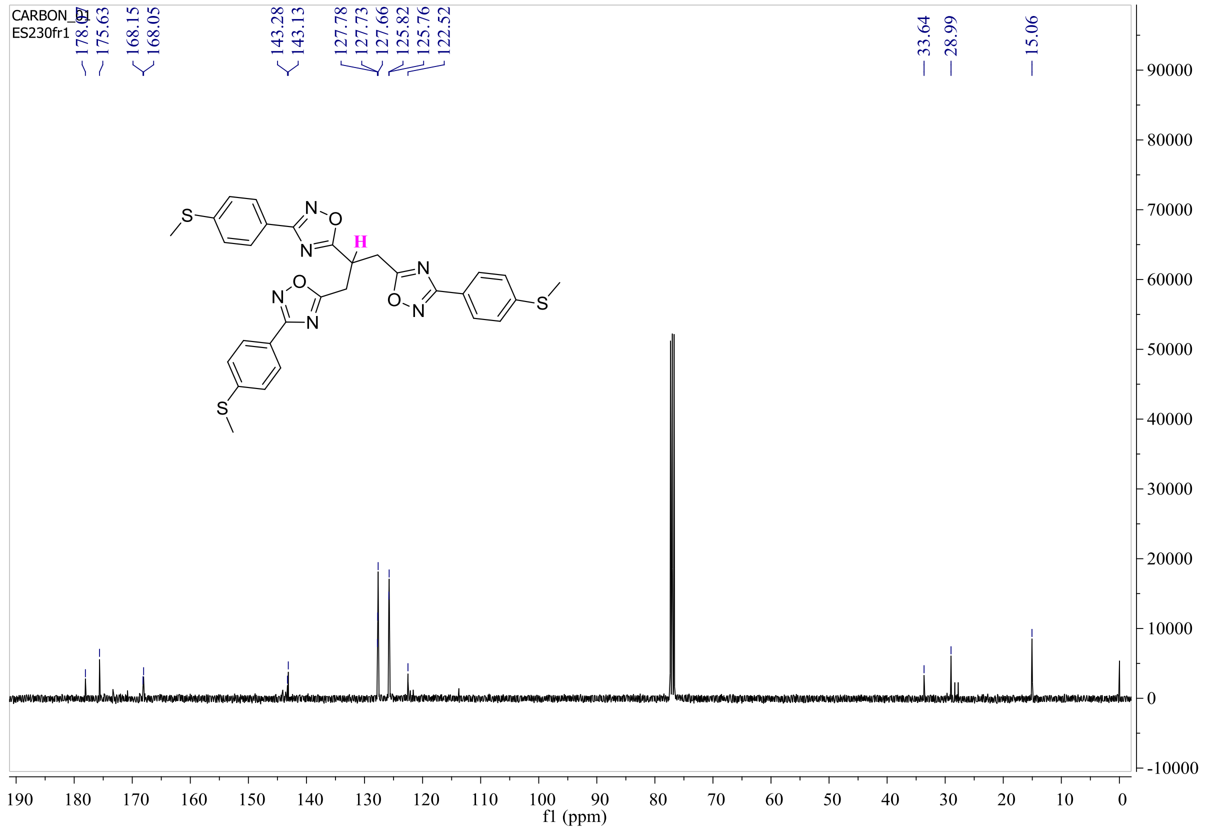

HSQC and HMBC SPECTRA of 3a

HSQC Spectrum of **3a**

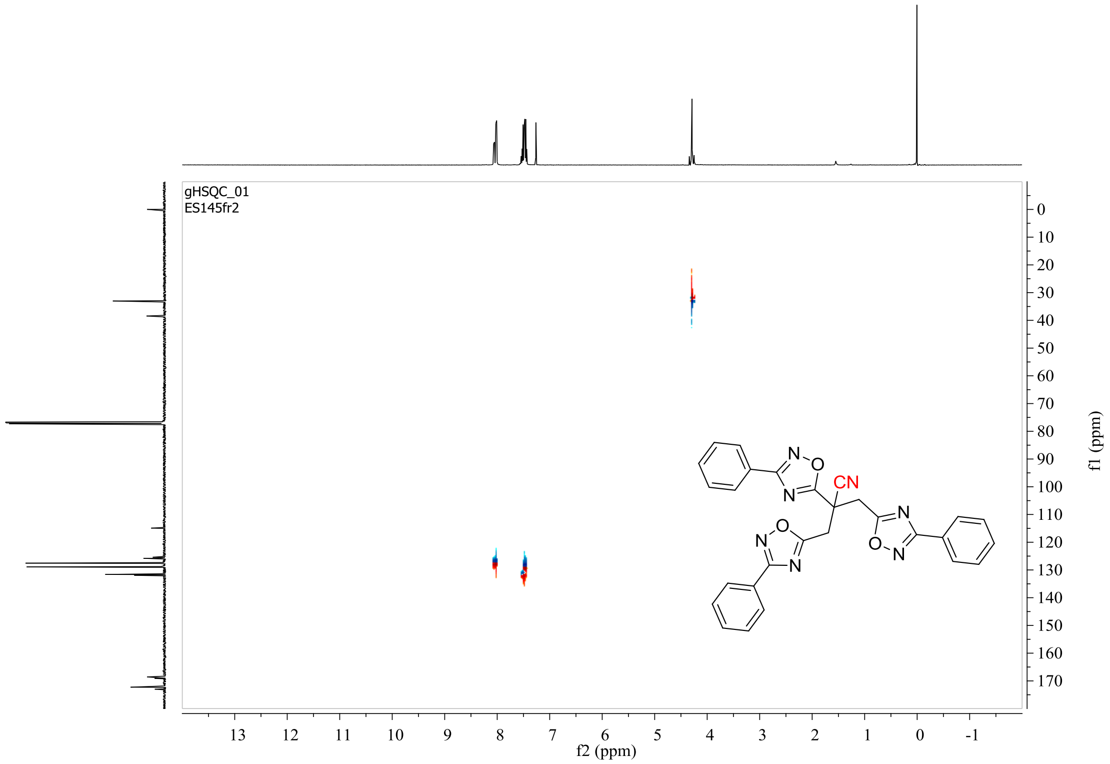

HMBC Spectrum of **3a**

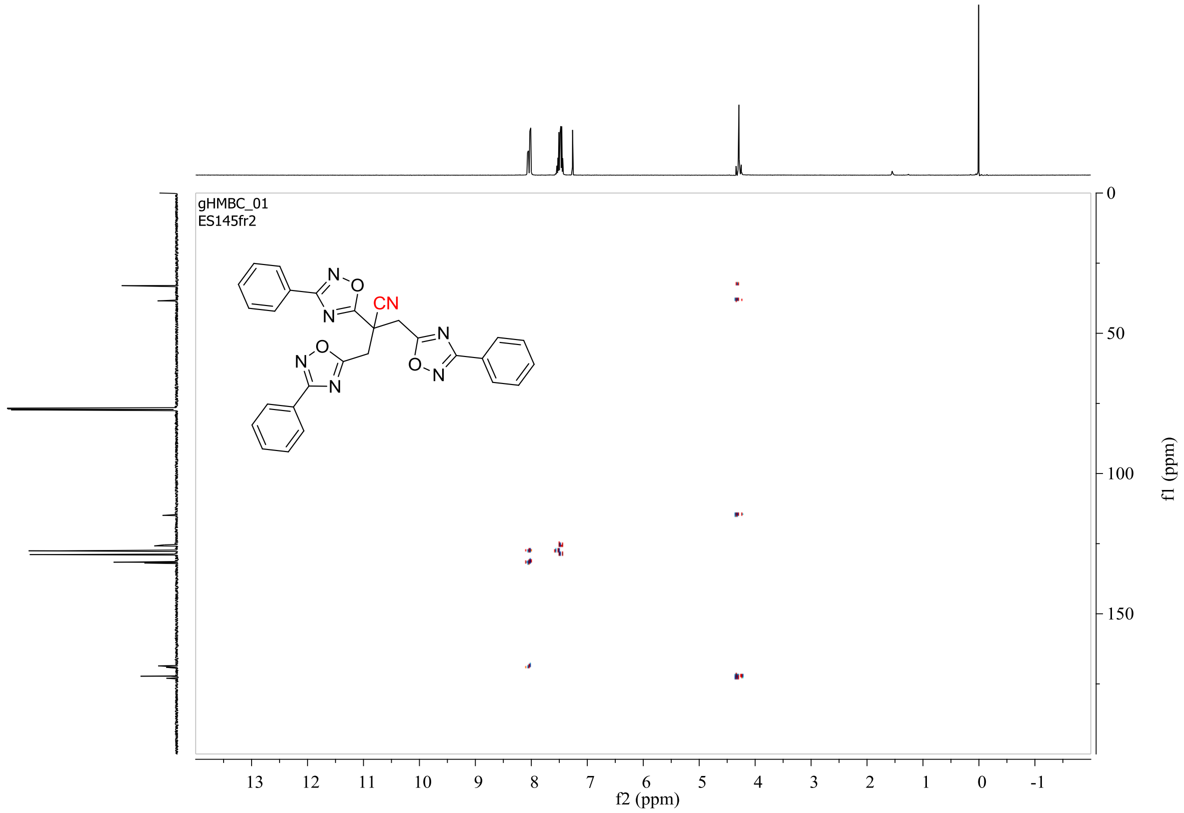

HSQC and HMBC SPECTRA of 4a

HSQC Spectrum of **4a**

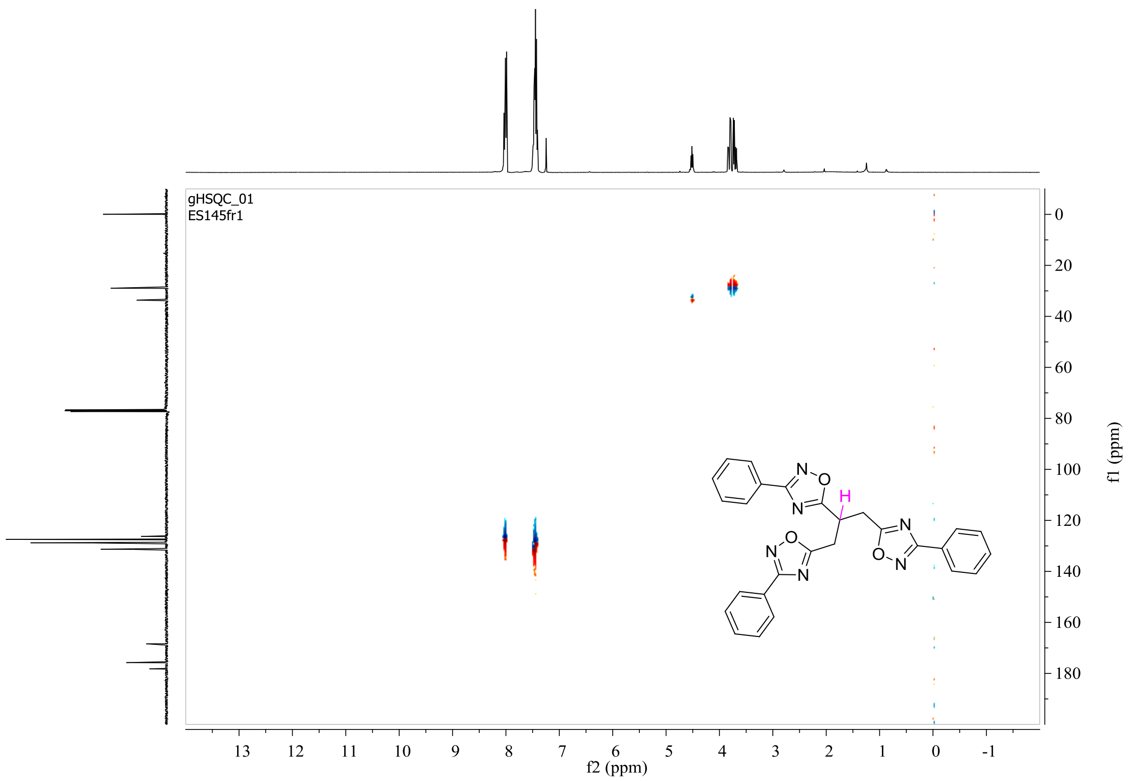

HMBC Spectrum of **4a**

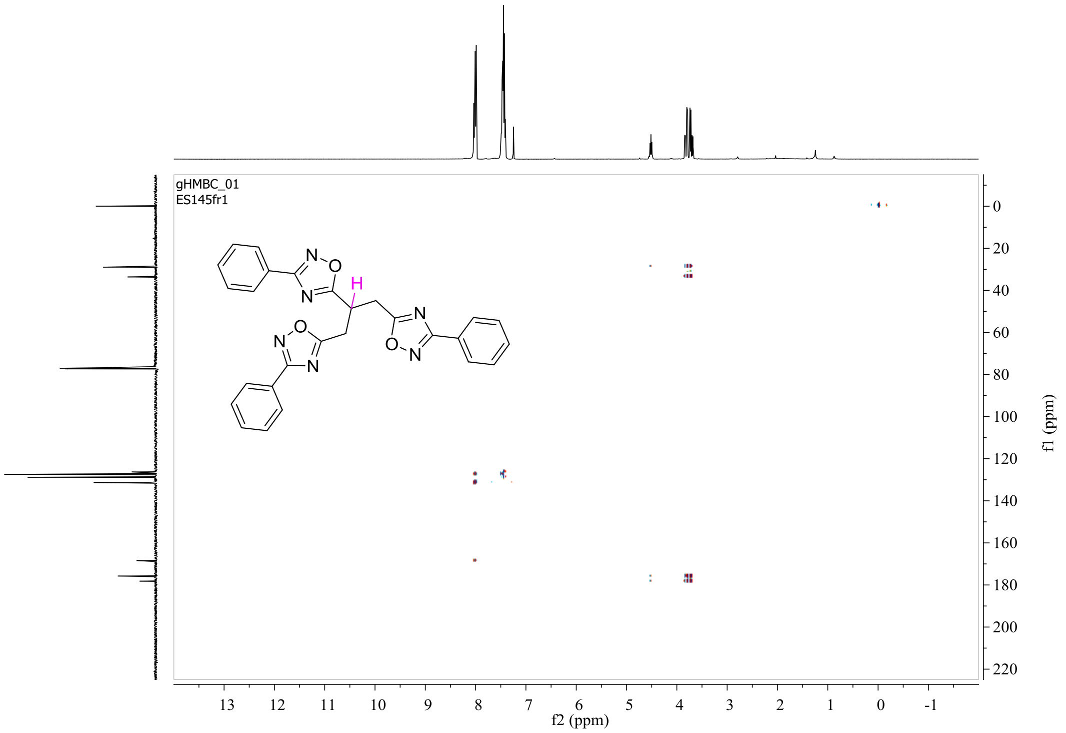

References

1- H. Ağırbaş, D. Sümengen, Y. Dürüst and N. Dürüst, *Synth. Commun*., 1992. **22**, 209-217.
